# Astrocytic IGF-1 and IGF-1R Orchestrate Mitophagy in Traumatic Brain Injury via Exosomal miR-let-7e

**DOI:** 10.1155/2022/3504279

**Published:** 2022-08-24

**Authors:** Ren Dabin, Chen Wei, Shu Liang, Cao Ke, Wang Zhihan, Zheng Ping

**Affiliations:** ^1^Department of Neurosurgery, Shanghai Pudong New Area People's Hospital, Shanghai, China; ^2^Department of Neurology, Shanghai Ninth People's Hospital, Shanghai, China

## Abstract

Defective brain hormonal signaling and autophagy have been associated with neurodegeneration after brain insults, characterized by neuronal loss and cognitive dysfunction. However, few studies have linked them in the context of brain injury. Insulin-like growth factor-1 (IGF-1) is an important hormone that contributes to growth, cell proliferation, and autophagy and is also expressed in the brain. Here, we assessed the clinical data from TBI patients and performed both in vitro and in vivo experiments with proteomic and gene-chip analysis to assess the functions of IGF-1 in mitophagy following TBI. We show that reduced plasma IGF-1 is correlated with cognition in TBI patients. Overexpression of astrocytic IGF-1 improves cognitive dysfunction and mitophagy in TBI mice. Mechanically, proteomics data show that the IGF-1-related NF-*κ*B pathway transcriptionally regulates decapping mRNA2 (Dcp2) and miR-let-7, together with IGF-1R to orchestrate mitophagy in TBI. Finally, we demonstrate that brain injury induces impaired mitophagy at the chronic stage and that IGF-1 treatment could facilitate the mitophagy markers via exosomal miR-let-7e. By showing that IGF-1 is an important mediator of the beneficial effect of the neural-endocrine network in TBI models, our findings place IGF-1/IGF-1R as a potential target capable of noncoding RNAs and opposing mitophagy failure and cognitive impairment in TBI.

## 1. Introduction

Traumatic brain injury (TBI) is one of the most common neurological diseases with higher mortality and morbidity annually [1]. A lot of TBI patients also develop cognitive dysfunctions [2]. However, there are still no effective treatments to improve cognition or promote its recovery in clinical sessions, and it is unclear which factor contributes to the cognitive recovery after TBI as well.

One potential factor might be posttraumatic hypopituitarism, which can be triggered by TBI and affect the process of brain repair [3, 4]. Insufficiency of growth factor (GH) is considered the major risk factor for TBI outcome, and insulin-like growth factor-1 (IGF-1) is a directly downstream of GH [5]. GH replacement in TBI increases serum IGF-I and reduces cognitive dysfunction [6]. IGF-1 variation has been associated with the intactness of brain structure and cognitive performance [7]. We previously demonstrated that astrocytic IGF-1 can prevent excitotoxicity in neurons and regulate the expression of GSK-3*β*, a kinase for tau phosphorylation, and thereby reducing the hyperphosphorylated tau.

IGF-1 has been shown to increase the mitophagy by activating adenosine 5′-monophosphate- (AMP-) activated protein kinase (AMPK) signaling [8]. We have shown that mitophagy is impaired in the chronic stage of brain injury [9, 10], and accordingly, we predict that this might be due to the lower IGF-1 status after TBI, as astrocytes have a supporting role in neurons and protect against excitotoxic injury in an IGF-1-dependent manner [11–13]. Although a series of studies have applied IGF-1 in brain insults such as stroke and TBI, the clinical application is limited due to its adverse effects found in animal studies. Therefore, it is important to explore the potential mechanisms in IGF-1-treated animals to identify new targets.

Here, we applied proteomics to study the therapeutic mechanisms in astrocytic IGF-1 after brain insults and further link the downstream pathway with mitophagy and neurological outcome. We found the enriched hydrolysis pathway, which may regulate the expression of miRNAs and impair mitophagy after TBI. Therefore, we hypothesized that IGF-1 status would affect mitophagy and cognitive recovery via miRNAs in TBI.

## 2. Methods

### 2.1. Patients and Serum IGF-1 Status

Human blood samples were obtained for analysis from TBI patients recruited in the Department of Neurosurgery at Shanghai Pudong New Area People's Hospital with institutional approval from the Ethics Review Board (Ethics No. 2170063) from Jan 2019 to Jul 2019. Venous blood (2-3 ml) from TBI was collected at 8 am on 3 days after injury and stored at -80°C until analysis. Absolute IGF-I was tested by radio autoimmune assay as previously reported [5]. Patients were then assigned to groups with different brain severity (Glasgow Coma Scale (GCS)), neurological outcome (Glasgow Outcome Scale (GOS)), and cognition scores (Montreal Cognition Assessment (MoCA)) to identify the relationship between IGF-1 and these parameters. To account for the findings that IGF-I level declines naturally with age, the groups were age-matched. There were no significant differences regarding the basic characteristics of patients and healthy controls (age, gender, education background). Blood collection and clinical data from all patients were obtained from their relatives with the consent form.

### 2.2. Single-Cell RNA-seq Data Processing

Single-cell transcriptome data of GSE160763 were downloaded from the Gene Expression Omnibus (GEO) database (http://www.ncbi.nlm.nih.gov/geo/). The chipset data from TBI patients was previously reported and used as an external verification here. R (v. 4.0.2) was used for bioinformatical analysis.

### 2.3. Single-Cell RNA-seq Analysis

The “Seurat” package was applied to carry out the sc-RNA-seq study. The dimension of data was reduced by PCA, uMAP, and t-SNE method. Marker genes for different cluster were identified by “Seurat” package as well. All clusters were annotated by “SingleR” package with a mouse dataset, and cell-cell communication was performed by “CellChat” package. Cluster biomarkers were found by the “Seurat” package. Monocle 3.0 was used to do the pseudotime analysis in interested cluster.

### 2.4. Cognition Assessment

Patients underwent a MoCA testing for cognitive assessment after TBI at six months post injury [14–16]. All TBI patients were assessed at 6 months post injury in our study as well. The total MoCA score is 30 points, and cognitive dysfunction is indicated by less than 26. The item of MoCA includes visuospatial and executive functioning, naming, attention and calculation, language, abstraction, delayed recall, and orientation.

### 2.5. Reagents and Antibodies

The rabbit polyclonal antibody pS356, which recognizes phospho-tau at Ser356, was purchased from Abcam (ab92682, Abcam, recombinant antibody). The mouse monoclonal antibody NeuN, which recognizes neurons in the brain, was also purchased from Abcam (ab104224, Abcam). Rabbit polyclonal anti-Dcp2 (ab28658, Abcam) and mouse monoclonal anti-IGF-1R (ab16890, Abcam) were purchased from Abcam. Rabbit polyclonal to ATG8 (ab4753, Abcam), mouse monoclonal to Atg5 (ab238092, Abcam), rabbit polyclonal to ATG14L (ab227849, Abcam), rabbit monoclonal (EPR19662) to Beclin-1, rat monoclonal (1D4B) to LAMP1 (ab25245, Abcam), and mouse monoclonal to SQSTM1/p62-autophagosome marker (ab56416, Abcam) were used to assess the mitophagy level in cultured cells. Mouse monoclonal (HM-2) to MAP2 (ab11267, Abcam) and rabbit monoclonal (EPR16778) to beta-tubulin (ab201831, Abcam) were used to evaluate the neuronal growth situation in vitro. Rat BNIP3 (ab10433, Abcam) and PINK1 (23274-1-AP, Proteintech) were used for mitophagy assessment in TBI mice; mouse monoclonal (mAbcam 8226) to beta actin was used as a loading control (ab8226). A bicinchoninic acid protein assay kit (BCA kit) was purchased from Pierce Biotechnology. An enhanced chemiluminescence detection kit was purchased from GE Healthcare. AG1024 (121767) was purchased from Sigma-Aldrich in the Inhibitor Select IGF Signaling Pathway Inhibitor Panel (407249, Sigma-Aldrich). LY294022 (#9901), an inhibitor of phosphatidyl-inositol 3-kinase (PI3K) and SB203580 and p38 MAPK inhibitor (#5633), was purchased from Cell Signaling Technology. MG-132, an Nfkb1 inhibitor (#474790), was purchased from Sigma (a knockdown assay was performed by transfection using validated siRNAs targeting NFKB1/p50 (SI02654932) from Qiagen). We purchased the negative control siRNA (AM4611) from Invitrogen company. We also consequently transfected nfkb1 and empty plasmid (pcDNA3, Life Technologies, Grand Island, NY) with FuGENE HD Transfection Reagent (Roche, Nutley, NJ).

### 2.6. Primary Cortical Neuronal Cultures and Coculture with Astrocytes

Primary culture of cortical neurons and astrocytes was previously reported. Cortical cultures were obtained from either E18. In brief, harvested cerebral cortex was digested in HBSS and resuspended in Neurobasal medium supplemented with 2% B-27 and 1% glutamine to inhibit the glial division. Cells were plated onto 6 dishes coated with poly-l-lysine (1 *μ*g/ml) at a final density of 1.5∗10^6^/well for neurons according to previous reports [12]. Excitotoxicity was introduced via kainic acid (KA) treatment of the neurons at 1 nmol/l cultured, and the coculture was performed with a Transwell device and supported with astrocytes collected from day 7 mice as described previously [12]. For the pharmaceutical manipulation, the cultured cells were treated with 1 *μ*M of IGF-1R inhibitor, 10 M of the PI-3 kinase/Akt inhibitor LY294022, and 10 *μ*M of p38 MAPK inhibitor SB203580.

### 2.7. Gene Transfer and Virus Constructs

Lateral ventricle injection of hIGF-1 for all animals was also reported previously with GFAP-AAV8-hIGF-1 or GFAP-AAV8-control, both having GFAP promoter to induce the specific expression of virus in astrocytes. All animals were given four-week recovery to allow the proper expression of virus after TBI or sham surgery [12]. The transfection efficiency of this virus and IGF-1 expression before and after virus injection have been validated in previous reports [12].

### 2.8. Downregulation of miR-let-7e and IGF-1R Inhibitor Assay

miR-let-7e antagonist (a synthetic double-stranded RNA oligonucleotide mimicking miR-let-7e precursor) and negative control (NC) as synthetic negative control RNAs were purchased from RiboBio (Guangzhou, China). 1 nmol miR-let-7e antagonist or NC in 5 *μ*l PBS was injected into the ventricle of TBI mice using a 30-gauge needle with 5 *μ*l Hamilton syringe injections. For in vitro studies, primary cultured neurons were seeded into 6-well plates and transfected the following day when the cells were approximately 70% confluent using Lipofectamine 2000 (Invitrogen, Carlsbad, CA, USA) according to the manufacturer's instructions. For each well, equal dose (1 nmol) of miR-NC or miR-let-7e antagonist was added. Cells were harvested 24 h after transfection, and total RNA was extracted for quantitative RT-PCR analysis and chip assay. The IGF-1R inhibitor (AG1024) was applied to cultured neurons to confirm the role of miR-let-7e.

### 2.9. Mouse TBI Model, qRT-PCR, Western Blot, and IHC

The detailed procedures were carried out as previously described [17]. Six- to eight-week-old male C57B6 mice were administered a lateral FPI as previously described [17]. These mice were obtained from our breeding colony in the Department of Medicine, University of Shanghai Medical Health, individually housed in 12 h light/dark cycles with food and water available ad libitum. We determined the sample sizes of rodents we used based on our previous reports [18, 19]. We used 10 rodents in each group randomly to minimize the number of animals we used in the experiments. All animal experiments were approved by the Animal Ethics Committees. The qRT-polymerase Chain Reaction (PCR) primers and antibodies are listed as informed (Supplementary Table 1). The raw WB blots were attached in Supp Figure 14.

### 2.10. iTRAQ Labeling

Each (isobaric tag for relative and absolute quantitation (iTRAQ)) reagent (Nr01, Nr02, Nr03, and Nr04 isobaric tags) was reconstituted in ethanol, and peptide samples were reconstituted in iTRAQ dissolution buffer (AB Sciex). Samples in sets of three biological replicates were labeled with the iTRAQ reagents as follows: sham+IGF-1 with Nr01 isobaric tag, sham+vector with Nr02 isobaric tag, TBI+IGF-1 with Nr03 isobaric tag, and TBI+vector with Nr04 isobaric tag. These samples were incubated at room temperature for two hours followed with centrifugations. The samples were further dried by vacuum centrifugation for approximately 1 h and stored at -20 Celsius degrees until liquid chromatography-mass spectrometry (LC-MS) analysis.

### 2.11. LC-MS Analysis

iTRAQ labeling was conducted according to the manufacturer's instructions (Zhonghong, Nanchang, China). Briefly, proteins from the four groups of rodent's brain were harvested, reduced, alkylated, and digested with trypsin overnight at 37°C before being labeled by iTRAQ. The labeled peptides were pooled and desalted with Sep-Pak Vac C18 cartridges (Zhonghong, Nanchang, China) followed by separation and analysis with a nano-Acquity ultra performance LC system (100 *μ*m~ 100 mm C18BEH column) (Zhonghong, Nanchang, China) coupled to a Q Exactive mass spectrometer (Thermo Fisher Scientific, Shanghai, China). All raw files were analyzed using the Thermo Proteome Discoverer (1.5.0) software. The files were searched using the Mascot (2.2.04) search engine against the UniProt human (72 390 entries) and decoy human databases with a strict false discovery rate (FDR) of 0.01. The fold change threshold was set at 2.0. A *p* value (*p* < 0.05) criterion (between TBI/sham ratios and IGF-1/vector ratios) was used to determine differentially expressed proteins.

### 2.12. Luciferase Reporter Assay

A luciferase assay was carried out as previous report [20]. Primary cultured neurons (3∗10^4^ cells/well) were selected in 96-well plates and permitted to incubate for one day. The indicated plasmids and 1.5 ng pRL-TK Renilla plasmid were transfected using Lipofectamine 3000 Reagent (Thermo Fisher Scientific, MA, USA). After 48 h posttransfection, luciferase signals were assessed by luciferase assay (E1980, Promega). The target site of IGF-1R and corresponding miR-let-7e binding was determined by online prediction software http://www.targetscan.org, and the primers were designed with the 3′-untranslated region (3′UTR) sequence of IGF-1R gene.

### 2.13. Exosome Isolation from Cell Cultures and NTA Analysis

Cortical cultures were obtained from either E18 or P3 rat (P3 days for astrocytes and E18 for neurons) as previously reported [11, 12]. The cultured medium from four groups Q1 ~ Q4 were collected and stored in 4°C fridge.

Exosome isolation: the supernatant of cell medium was taken from the 4°C freezer, balanced with PBS, and centrifuged at 1500*g*, 4°C for 30 minutes; the supernatant was taken and for a next 10000*g*, 4°C, centrifugation for 60 minutes; after this, the supernatant was centrifuged again at 12000*g*, 4°C for 30 minutes; next, the centrifugated supernatant was carefully moved to a single-use ultracentrifugation, at 110000*g* for 60 minutes at 4°C; carefully discard the supernatant after centrifugation, and the trace liquid at the bottom of the obtained centrifuge tube is exosomes. Carefully blow the bottom of the centrifuge tube with 30-100 *μ*l volume of 1×PBS and inhale into 0.5-1.5 ml centrifuge tube, gently pipet with a pipette to completely dissolve.

For the transmission electron microscopy morphology investigation, 10 ml of exosome pellet was placed on formvar carbon-coated 200-mesh copper electron microscopy grids and incubated for 5 min at room temperature and then was subjected to standard 1% uranyl acetate staining for 1 min at RT. The grid was washed with three times of PBS and allowed to semidry at room temperature before observation in transmission electron microscope (Hitachi H7500 TEM, Japan).

Analysis of absolute size distribution and concentration of exosomes were determined using nanoparticle tracking analysis (NTA). Exosomes were diluted in 1 ml PBS and mixed well; then, the diluted exosomes were injected into the NanoSight NS300 instrument (Malvern, UK); particles were automatically tracked and sized based on Brownian motion and the diffusion coefficient. Filtered PBS was used as controls. The NTA measurement conditions 25 frames per second, measurement time 60 s. The detection threshold was similar in all the samples. Three recordings were performed for each sample.

### 2.14. miRNA Quantitative RT-PCR (qRT-PCR) Array

The miRNA qRT-PCR array experiments were conducted at Wcgene Biotechnology Corporation, Shanghai. Total RNAs, including miRNAs, were isolated from 100 *μ*l of liquid sample, using a 1-step acidified phenol/chloroform purification protocol. Synthesized exogenous RNAs were spiked into each sample to control for variability in the RNA extraction and purification procedures. The purified RNAs were polyadenylated through a poly (A) polymerase reaction and were then reversed-transcribed into cDNA. Individual miRNAs were quantified in real-time SYBR Green RT-qPCR reactions with the specific MystiCq miRNA qPCR Assay Primers (Sigma-Aldrich). The protocol of miRNA qRT-PCR array analysis was as described in detail on the website of Wcgene (http://www.wcgene.com).

### 2.15. Chromatin Immunoprecipitation-qPCR (ChIP-qPCR)

Chromatin immunoprecipitation (ChIP) was carried out according to the manufacturer's protocol. First, after crosslinked with 1% formalin, cells were seeded in SDS buffer. Nuclei were pelleted and resuspended in IP buffer (2 volumes SDS lysis buffer:1 volume Triton-X buffer (100 mM Tris-Cl, pH 8.6, 100 mM NaCl, 5 mM EDTA pH 8.0, 5% Triton X-100)). The lysates were sonicated using Bioruptor sonicator for 12 cycles of 30 s and centrifuged at maximum speed. ChIP for decapping enzyme homolog (DCP2) was carried out using a Flag antibody (Sigma, SAB4301135). Eluted DNA fragments were further purified using Minelute PCR Purification Kit (Qiagen) and then amplified by qPCR. The primers are listed in Table S1.

### 2.16. Dcp2 Knockdown Assay

To investigate the altered genes in Dcp2 deficiency neurons, we applied RNAi method to reduce the expression of Dcp2 and further looked at the changed mRNAs and miRNAs with a customed chipset. Then, the ratio of Dcp2 KO and WT was listed, and the heat map was produced.

### 2.17. JC-10 Staining

We applied flow cytometry to evaluate the mitochondrial membrane potential with the fluorogenic dye, JC-10 (Abcam 112133). Cells seeded with JC-10 were added at an equal volume and incubated in the dark room at 37°C for 15 minutes prior to analysis. Monomeric (green) and J-aggregate (red) fluorescences were, respectively, measured using the Flow1 and Flow2 channels. The results were analyzed in six groups as mentioned above.

### 2.18. Assessments of Motor Function and Cognitive Performance

Motor function was evaluated at 1, 3, 5, and 7 days after TBI using the neurological severity score (NSS) method [21]. Briefly, it includes forelimb flexion, lateral push, forelimb and hindlimb placement, vestibulomotor function, and motor performance on a balance beam: score of 0–1 or 0–2 for neuromuscular functions, 0–6 for vestibulomotor functions, and 0–5 for complex neuromotor functions.

For the Morris water maze (MWM), the water temperature was maintained at 25 ± 1°C. To ensure motor recovery, tests were carried out two weeks post-TBI. A minimum of 5 minutes was set between two trials. The latencies for the mouse to locate the platform and time duration in other zones were recorded and analyzed using a tracking device (Noldus 2.0, Netherlands).

### 2.19. Statistical Analysis

The results are reported as the mean ± SD for immunoblots, fluorescence experiments, and PCR results. Gray levels were detected with the ImageJ software (Oxford, UK). One-way ANOVA was used to compare the differences among groups with the Prism software 8.00 (GraphPad, Prism). For behavioral tests, data were expressed as the mean ± SD. Motor and MWM data, which are continuous, were analyzed by repeated ANOVA, followed by Tukey's post hoc test for between-group comparisons. *p* < 0.05 was set as statistical difference.

## 3. Results

### 3.1. Serum IGF-1 Is Reduced after TBI

**Figure 1:**
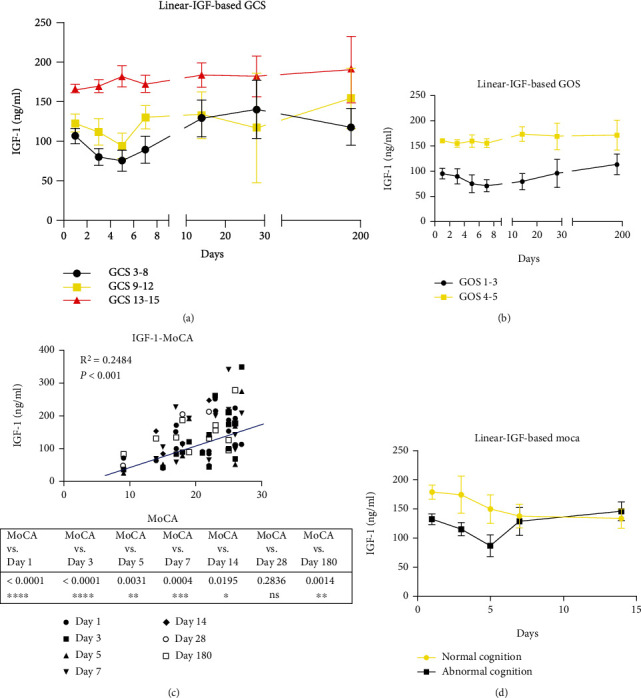
The correlation between dynamic serum IGF-1 and GCS and GOS and MoCA. (a) Serum IGF-1 is lower in patients with mild TBI than those with severe TBI. (b) Serum IGF-1 in the good outcome group (GOS4-5) is higher than in the poor outcome group (GOS1-3). (c) Serum IGF-1 is positively associated with MoCA score (*R*^2^ = 0.775, *p* < 0.05). (d) Serum IGF-1 in the normal cognition group is higher than in the abnormal cognition group. The data was expressed as mean ± SD.

Serum IGF-1 is lower in patients with TBI than healthy controls; the serum IGF-1 content in moderate and severe TBI at each time point was significantly lower than that of the mild TBI group (*p* < 0.05). There is no difference regarding the age at onset, gender distribution, and time since injury for IGF-1 test (Table 1). Serum IGF-1 progressively decreased after TBI in the first week, while it began to increase on the seventh day, but it remained higher than the control group at six months post injury (*p* < 0.05, Figure 1). Meanwhile, we found that patients with lower IGF-1 had more severe injury reflected by pituitary deficiency, structural brain abnormality, and more contusions (Table 1).

Serum IGF-1 protein was significantly higher in the poor outcome group (GOS 1-3) that in the good outcome group (GOS 4-5, *p* < 0.05; Figure 1). Serum IGF-1 content at 1, 3, 5, 7, and 14 days after injury (acute and subacute stage) had a significant positive correlation with MoCA score even at 6 months after injury (*R*^2^ = 0.2484, *p* < 0.001). Serum IGF-1 in the normal cognition group was higher than that in the abnormal cognition group at the acute stage of TBI (Figure 1).

### 3.2. IGF-1 Is Altered in TBI from sc-RNA-seq Data

All cells obtained from GSE 160763 (sc-RNA-seq data) were grouped into 21 clusters with UMAP. The 21 clusters were further annotated with SingleR package as listed (Supp Figure 1A). We then listed the expression of marker gene IGF-1 in different clusters (Supp Figure 1B&C). We further looked at the cell-cell interaction based on the IGF-1 with “CellChat” R package.

We found that the IGF-1 signaling is mainly occurred among clusters 4, 9, 13, and 20 (Supp Figure 1A-C). Further, we showed that clusters 13 and 15 as a sender and clusters 4 and 9 as a receiver with a focus on IGF-1 (Supp Figure 2A-D). As IGF-1 is a marker gene mainly located in cluster 15 and both clusters 15 (astrocyte) and 9 (neurons) are most enriched clusters, we further investigated their interaction with a pseudotime analysis tool.

With the “Monocle 3.0” R package, we found that there was a development trend from cluster 15 to cluster 9 for IGF-1 (Supp Figure 2E-H). The violin map showed the expression of marker genes, Igf-1, Igf-2, and Igf1r in different clusters, which is consistent with the cell-cell interaction model (Supp Figure 2I).

### 3.3. Astrocytic IGF-1 Is Neuroprotective in TBI Models

**Figure 2:**
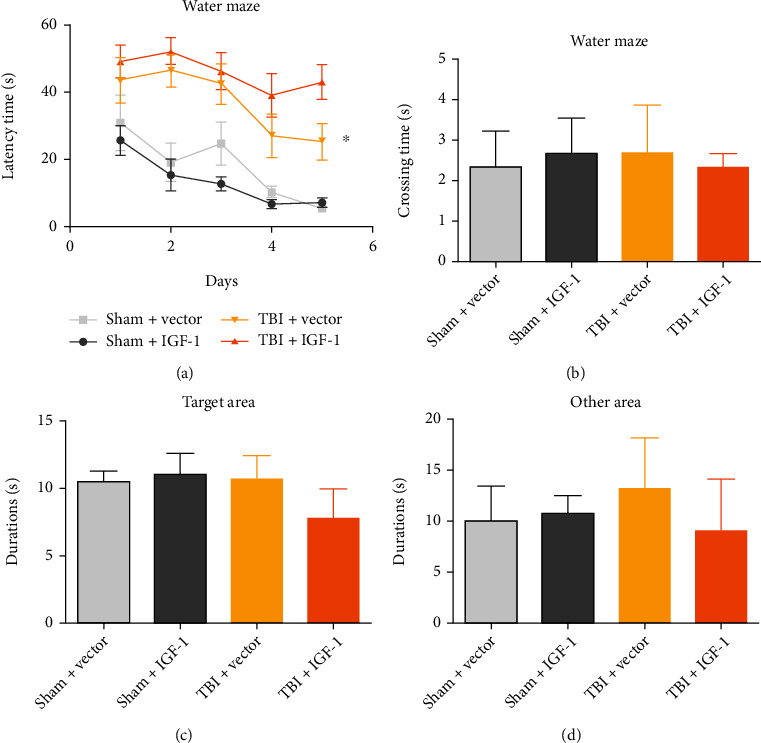
Astrocytic IGF-1 reduces cognitive and motor impairments after TBI. (a) Mice given an FPI and treated with vector had longer search times in the water maze, whereas TBI mice treated with astrocytic IGF-1 displayed fewer search times. (b) There was no difference in crossing time or duration among the groups. (c, d) Regarding motor function, TBI mice with vectors have higher NSS scores, and astrocytic IGF-1 could reduce NSS. ^∗^The TBI groups are significantly different than the sham groups, *p* < 0.05. *n* = 10 in each group, and the data was expressed as mean ± SD.

Previously, we showed that astrocytic IGF-1 protects against excitotoxic neurons both in vivo and in vitro. Therefore, we used astrocytic IGF-1 to treat a lateral fluid percussion injury (LFPI) mouse model. For MWM test, FPI mice treated with astrocytic IGF-1 showed much lower latency time than vehicle-treated mice during the acquisition session. There were no significant differences in crossing time or duration in the targeted zone during the water maze acquisition period (*p* > 0.05). Furthermore, TBI mice had higher NSS, while astrocytic IGF-1 could reduce it, which indicates that astrocytic IGF-1 improved both cognition and motor function in TBI mice by the Morris water maze and NSS assessment (Figure 2).

### 3.4. iTRAQ and Bioinformatic Analysis in IGF-1-Treated TBI Mice

**Figure 3:**
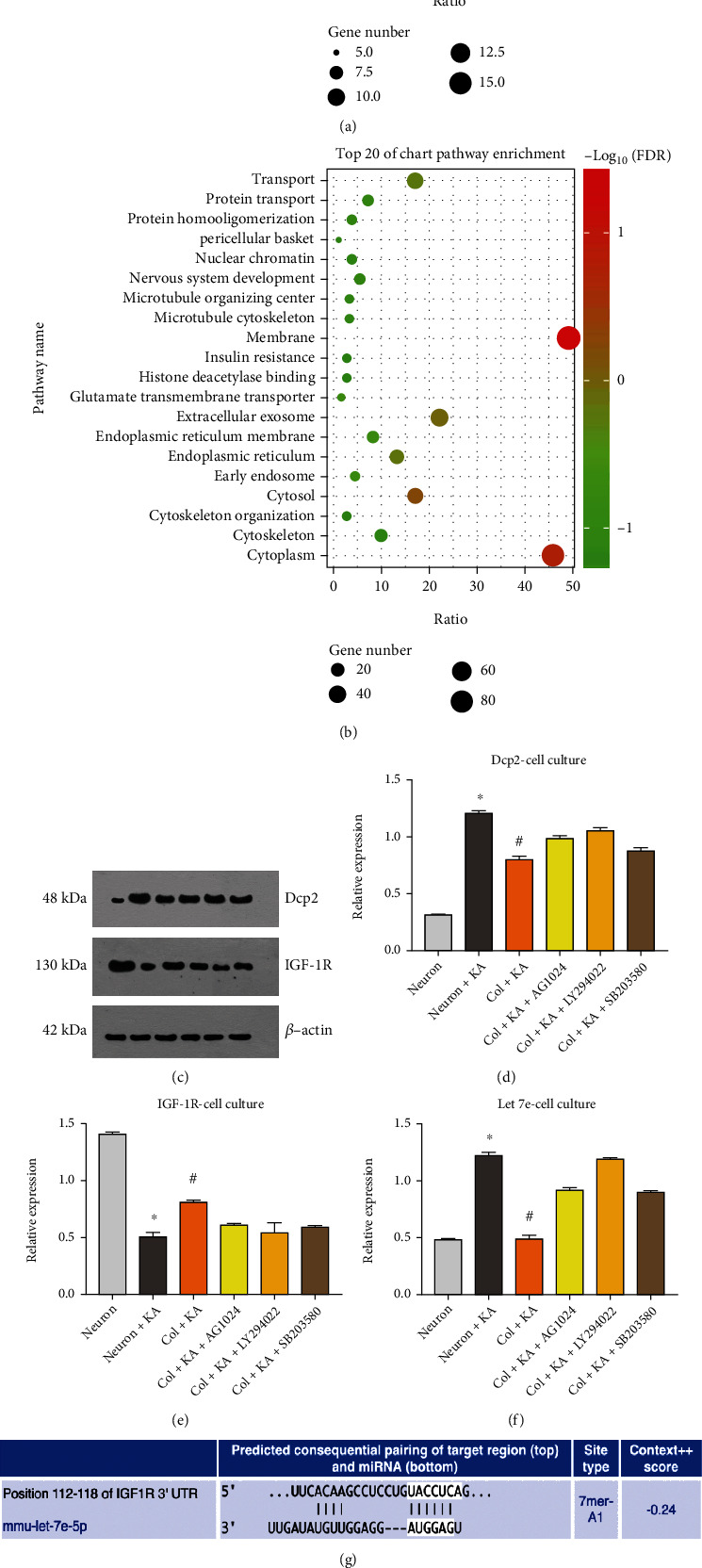
Pathway enrichment shows hydrolase activity and Dcp2 changes after brain insults (a, b). An in vitro study showed that Dcp2 and miR-let-7e increase in KA-treated neurons, and cocultured astrocytes could reduce these with IGF-1 pathway dependence (c, d). Brain IGF-1R decreases in KA-treated neurons, and cocultured astrocytes could reverse this (e). IGF-1R was also identified as a target gene of miR-let-7e. Putative miR-let-7e binding sites in IGF-1R 3′-UTR. Luciferase activity of IGF-1R-wt 3′-UTR after transfection with miR-let-7e mimic (f–h); ^∗^*p* < 0.05 compared with cotransfection of IGF-1R-wt and miR-let-7e NC. NC: negative control; wt: wild type; mut: mutation. ^∗^KA neurons vs. neurons, *p* < 0.05; ^#^versus KA neurons, *p* < 0.05. *n* = 3 in each group, and the data was expressed as mean ± SD.

Next, we applied iTRAQ proteomics to investigate the neuroprotective effect of astrocytic IGF-1. According to the iTRAQ method published previously, we compared the targeting proteins with a twofold alteration in the TBI vs. sham group with vectors and TBI+IGF-1 vs. TBI+vector. We identified 189 candidate proteins after IGF-1 treatment. First, we performed a DAVID analysis with an online tool (https://david.ncifcrf.gov) and clustered these candidate proteins based on KEGG analysis (Supp Figure 3). We listed the top 20 clusters and KEGG pathways in Figure 3. The top 20 cluster pathway (DAVID analysis) showed that hydrolase activity (GO:0016787~hydrolase activity) was the first pathway, including 16 related proteins (Q9CYC6, Q9CR30, Q50L41, Q9R001, Q6NSR8, Q8CGB6, Q9WUZ9, Q9ET22, Q91ZX6, P35821, P52479, Q8CHE4, Q9CXY9, P97470, P07146, Q14BV6).

### 3.5. DCP2 and Related Pathway Changes after TBI

DCP2 (Q9CYC6) was found to increase in the TBI group by more than 2-fold, and IGF-1 treatment reduced its expression level by 1.4-fold. Dcp2 encodes m^7^GpppN-mRNA hydrolase in mammals [22]. M^7^G is a 5′ capping structure in mRNA and in miRNA. METTL1 (another m7G decapping enzyme) has been found to promote the maturation of several miRNAs by disrupting the immature structure of miRNA [23]. And METTL1 knockdown could reduce the expression of a serious of miRNAs including let-7e with a most decreased level [23]. Here, we predicted that DCP2 is supposed to be decapping the m^7^G structure of let-7e as well as to promote its maturation and checked the expression of DCP2 and IGF-1R in cell models.

To validate this, we performed an in vitro analysis with an excitotoxic model that can mimic brain injury in cells. First, we observed that KA-treated neurons had decreased MAP2 and beta-tubulin expression, indicating reduced maturation of neurons, while coculture with astrocytes could promote neuronal growth and inhibit the excitotoxic effect with increased expression of both MAP2 and beta-tubulin to validate our previous findings and the successful modeling of in vitro excitotoxic injury (Supp Figure 4). Next, we found that both DCP2 and let-7e expression increased in KA-treated neurons, while coculture with astrocytes reduced both. These findings were consistent with our TBI in vivo results. This effect was abolished partly by the IGF-1R antagonist, PI3K inhibitor, and p38 inhibitor (Figure 3). Furthermore, we validated the relationship between Dcp2 and miRNAs with a Dcp2 knockdown method. We found that Dcp2 deficiency in neurons shows altered both miRNAs and mRNAs and mir-let-7e listed at the top downregulated miRNA in Dcp2 knockdown neurons (Supp Figure 5).

miR-let-7e has previously been reported to regulate IGF-1R [24]. In our study, we performed a TargetScan analysis to predict the binding between the IGF-1R 3′UTR and let-7e. Furthermore, we confirmed this with a luciferase assay. Next, the dual luciferase reporter gene assay with the psicheck2-based IGF-1R-wt plasmid containing the miR-let-7e binding site was conducted to further verify this prediction. The activity of the luciferase following cotransfection of IGF1R-wt and miR-let-7e mimic was lower than the activity following cotransfection of IGF-1R-wt and NC (*p* < 0.05), indicating that there was a regulatory relationship between miR-let-7e and the 3′-UTR of IGF-1R (Figure 3). These findings helped to verify that miR-let-7e targeted IGF-1R.

### 3.6. Astrocytic IGF-1 Regulates Mitophagy of Brain Injury In Vitro and In Vivo

**Figure 4:**
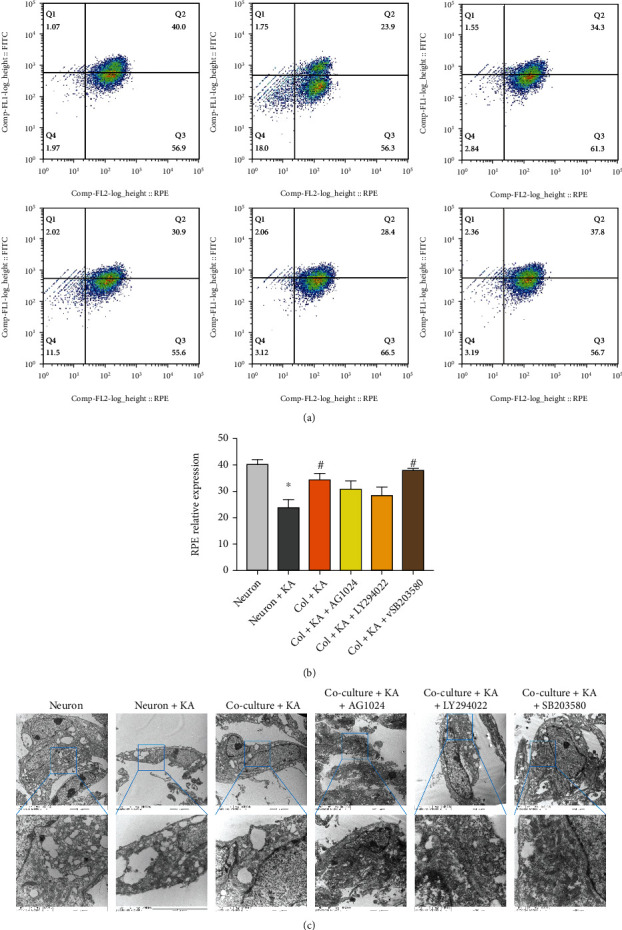
Cocultured astrocytes repress mitochondrial membrane disruption and mitophagy in an IGF-1-dependent manner. KA-treated neurons showed decreased mitochondrial membrane potential indicative of decreased Q2 (RPE level), while cocultured astrocytes could increase this. This effect was partly abolished by IGF-1R and PI3K inhibitors but not p38 MAPK inhibitors (a, b). ^∗^KA neurons vs. neurons, *p* < 0.05; ^#^versus KA neurons, *p* < 0.05. *n* = 3 in each group, and the data was expressed as mean ± SD. The electro image also shows that IGF-1 reverses mitophagy in KA-treated neurons. *n* = 1 in each group. The parameter for EM images including Mic type, HV value, magnitude level, and the scale bar was demonstrated in the original images as supplementary files.

IGF-1R can regulate autophagy, and IGF-1 can promote mitophagy by activating AMPK [8]. To confirm the role of IGF-1 in mitophagy following brain injury, we performed both in vitro and in vivo analyses of mitophagy. This study further evaluated whether the increased neuronal loss in KA-stressed cells was accompanied by a loss of mitochondrial potential and whether astrocytic IGF-1 could affect this phenomenon. The cells were analyzed with JC-10 dye, which forms red J-aggregates in controlled primary cultured cells but stays a green monomer in cells that have lost mitochondrial integrity. The scatter plots show that the majority of the cells treated with KA shifted towards green fluorescence when compared to controls (Figure 4). Remarkably, in KA-treated neurons cocultured with astrocytes, a population shift to the red channel was observed, indicating preservation of mitochondrial potential. In addition, this effect was partly abolished by the IGF-1R antagonist, PI3K inhibitor, and p38 inhibitor. Consequently, KA-stressed cells maintained mitochondrial potential when cocultured with astrocytic IGF-1 (Figures 4(a) and 4(b)). Accordingly, we further looked at the role of IGF-1 in mitophagy. We found that the mitophagy was impaired in neurons treated with KA and reversed by coculture with astrocytes (Figure 4(c)). However, this neuroprotective effect was partly abolished by IGF-1R antagonist (AG1024) and PI3K inhibitor (LY294022), but not with p38 inhibitor (SB203580). These results indicated that IGF-1 pathway is involved in mitophagy after cell injury.

**Figure 5:**
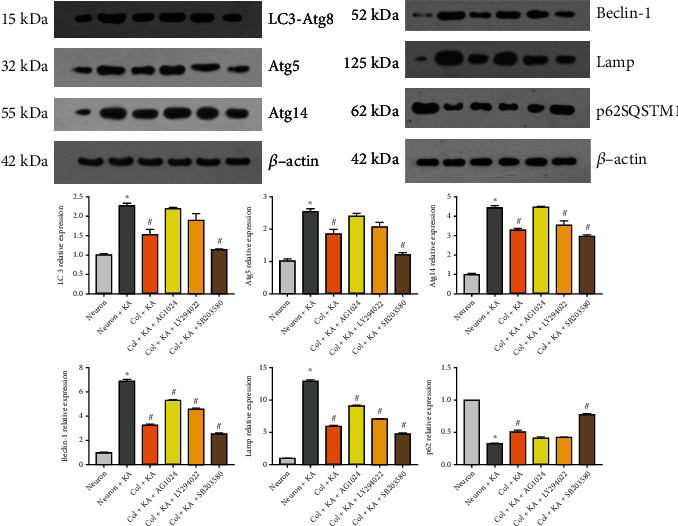
Activation of autophagy by KA. Expression of LC3/Atg8, Atg5, Atg14, Beclin-1, and Lamp1 increased in primary neurons treated with KA (1 *μ*M). The expression of p62 decreased after KA. Both changes are reversed by cocultured astrocytes, and this effect is differently abolished by AG1024, LY294022, and SB203580. *β*-Actin was used as a loading control. ^∗^KA neurons vs. neurons, *p* < 0.05; ^#^versus KA neurons, *p* < 0.05. *n* = 3 in each group, and the data was expressed as mean ± SD. WB: the left panel shared the same internal loading control, and the right panel also shared the same control.

In addition, we extracted mitochondrial proteins from cultured cells to assess mitophagy following KA. We found that KA increased the expression of Atg8, Atg5, Atg14, Beclin-1, and Lamp while decreasing the p62SQSTM1 level. This effect is partly abolished by AG1024, a PI3K inhibitor and a p38 MAPK inhibitor (Figure 5). The raw blots for Figure 5 are attached as Supplementary Figure 14.

**Figure 6:**
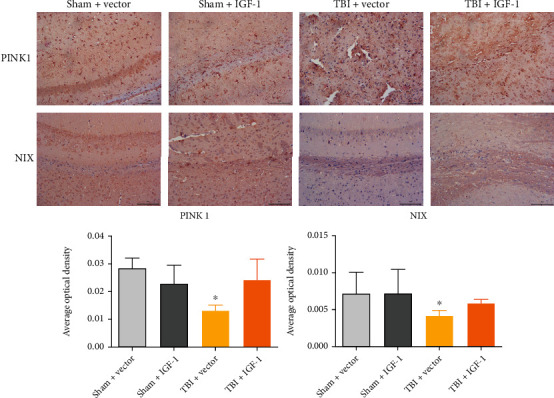
Dysregulated mitophagy in the chronic stage of TBI. Protein expression of PINK1 and NIX was reduced at three months post injury, and IGF-1 treatment increased both. ^∗^*p* < 0.05, compared to other groups. *n* = 3 in each group, and the data was expressed as mean ± SD. Bar = 200 *μ*m.

For the in vivo study, we applied IHC to analyze mitophagy markers (PTEN-induced putative kinase 1, PINK1 and Nip-like protein X, NIX) in the rodent TBI brain. As shown in Figure 6, FPI in mice induced decreased NIX and PINK1 expression (shown as the average optical density of the brown area) in the ipsilateral cortex of TBI mice at three months after injury, and IGF-1 treatment reversed these effects.

### 3.7. Astrocytic IGF-1 Alters Exosomal miR-let-7e in TBI

**Figure 7:**
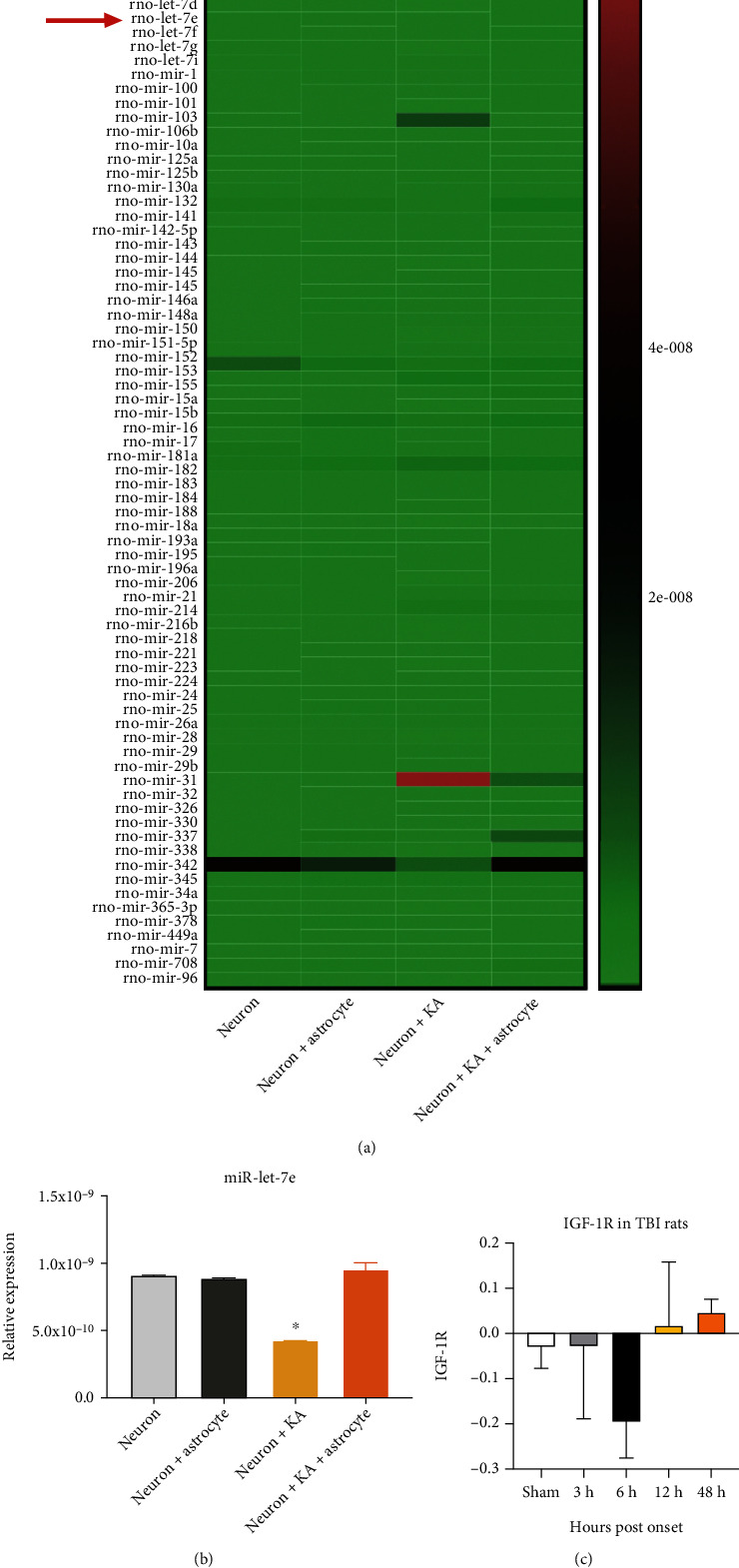
IGF-1 mediates the secretion of exosomal miR-let-7e, and let-7e inhibitor shows neuroprotection in TBI. (a) The heat map demonstrates the miRNA alterations in exosomes from primary cultured neurons, neurons cocultured with astrocytes, KA-treated neurons, and KA-treated neurons cocultured with astrocytes. (b) The expression of miR-let-7e, which was decreased in KA-treated neurons and the cocultured astrocytes could increase its expression. *n* = 3 in each group, and the data was expressed as mean ± standard deviation. (c) The expression level of IGF-1R in TBI rats obtained from GEO dataset (*x*-axis is time postonset; *y*-axis is the relative expression level).

We previously showed that TBI could promote the excretion of exosome [25] and IGF-1 treatment would reduce the expression of several miRNAs [26] including miR-let-7e [26]. We currently, among others, show that astrocytic-derived exosomes can be taken by neurons to carry out their neuroprotective roles after brain insults [27]. Here, we further confirmed this by exosome miRNA chipset in in vitro study. We found that KA treatment could increase the secretion of exosomes in the neuronal cultured medium (Supp Figure 9–11). And KA treatment would decrease the expression of miR-let-7 in neuronal exosomes (Figures 7(a) and 7(b)), which would be prevented by astrocyte coculture. The expression level of target gene (IGF-1R) of miR-let-7e reduced in the acute stage of TBI and recovered to the baseline after 12 hours (Figure 7(c)).

## 4. Discussion

In the present study, we applied iTRAQ proteomics to demonstrate a subset of proteins changed in TBI and reversed this change by genetic manipulation of astrocytic IGF-1 and miR-let-7e inhibitor. The pathway analysis showed that hydrolysis lied at the top in the cluster. We identified Dcp2, an m7G hydrolysis enzyme increased after TBI and decreased by IGF-1 treatment. Furthermore, we found that astrocytic IGF-1 could also reverse the interrupted mitophagy after TBI. A mechanical study demonstrated that Dcp2 could promote the maturation of the let-7 family and further decrease IGF-1R and related mitophagy. Based on these findings, we proposed an orchestration between IGF-1 and IGF-1R to facilitate the mitophagy after TBI via exosomal miR-let-7e.

### 4.1. A Decapping Mechanism between DCP2 and miR-let-7e

IGF-1/IGF-1R and miR-let-7e have been previously reported to downregulate each other and modulate proliferation and migration in colorectal cancer cells. The author also found that let-7e could reduce the expression of IGF-1R with qPCR test, and IGF-1 treatment was able to decrease the expression of let-7e as well. However, the authors did not further investigate the mechanisms [28]. In our report, we applied a luciferase assay to link let-7e and IGF-1R and a proteomic method to identify the mechanism behind how IGF-1 reduced let-7e with an m7G decapping mechanism.

The presence of m7G in miRNAs strongly indicates a new RNA methylated regulation in noncoding RNAs. This might exist in long noncoding RNAs (lncRNAs). A previous study identified that the G-quadruplex structure is known to be inhibitory to miRNA processing [29], and the G-quadruplex motif in let-7e overlaps with the DROSHA cleavage site. Therefore, we proposed that Dcp2-mediated deposition of m7G within G-rich regions destabilizes G-quadruplexes, thereby promoting their processing from pri- to pre-miRNA (Supp Figure 7). Geisler et al. reported that many lncRNAs are degraded by DCP2 [30], and a recent article has identified the catalytic structure of DCP2 [31]. It has also been reported that the DCP2 decapping enzyme is redundant and required for miRNA-mediated gene silencing [22]. Consistent with our studies, DCP2 might facilitate the maturation of miRNA to carry out the gene silencing function. More importantly, the DCP2 level is thought to affect the let-7 expression [32].

Removal of the 5′ cap on mRNA by the decapping enzyme Dcp2 is a critical step in 5′-to-3′ mRNA decay. However, a recent report found that the m7G methylation might promote miRNA processing with methyltransferase 1 (METTL1), a major methyltransferase. Thus, Dcp2 could decay mRNAs by facilitating their associated miRNA. The accumulation of miRNA targets increased concomitantly with the decrease in miRNA in the decapping mutants [33]. Therefore, we proposed that the increased Dcp2 after brain injury could facilitate the expression of miRNA and reduce the targeted mRNAs. Geisler et al. reported that many lncRNAs are degraded by DCP2 [30], and a recent article has identified the catalytic structure of DCP2 [31].

### 4.2. IGF-1 Regulates miRNA Expression through m7G Hydrolysis

Previous studies have shown that IGF-1 can regulate exosome-mediated miRNA transfer and maintain tumor cell proliferation [34]. IGF-1 from tumor-initiating cells can prevent miR-122 expression in neighboring normal hepatocytes and thereby facilitate its intercellular transfer within exosomes, leading to low levels of antiproliferative miRNAs in hepatic cancer cells. However, the author did not investigate the mechanism by which IGF-1 regulated miR-122 expression [35]. This finding is consistent with our study, and we further found that IGF-1 could regulate the miRNA expression through m7G hydrolysis with our proteomics data. Another study showed that macrophages can release IGF-1, influencing its phagocytic ability and inflammation. IGF-1 from macrophages can bind to the surface of nonprofessional phagocytes (such as epithelial cells). Meanwhile, IGF-1 could enhance the uptake of macrovesicles by epithelial cells, and deletion of the IGF-1 receptor led to exacerbated inflammation [36]. The similar cell-cell interaction was found in our single cell RNA-seq results as well (Supp Figure 1&2). Therefore, it would be interesting in future study to investigate how astrocytic IGF-1 influences exosome release and neuronal phenotype. In addition, exosomes are full of noncoding RNAs, including both miRNAs and lncRNAs, which could also be indirectly regulated by IGF-1.

### 4.3. IGF-1 Facilitates Cellular Proliferation through Mitophagy

As previously reported, exogenous IGF-1 sustains cell viability in cancer cell lines by stimulating mitochondrial biogenesis and BCL2 protein-interacting protein 3-like- (BNIP3-) induced mitophagy [37], and IGF-1 can also augment mitochondrial function and neuronal metabolism via AMPK [8]. However, no previous study has linked IGF-1 and neuronal mitophagy in brain insults. As tau phosphorylation can further impair the autophagy pathway and astrocytic IGF-1 could reduce phosphor-tau both in vivo and in vitro [12] (Supp Figure 6), we proposed that IGF-1 could promote the mitophagy and downregulate p-tau in a beneficial circle. One of the major pathological hallmarks of TBI is phosphorylated tau [18, 19], which results in poor clinical outcomes after TBI. We currently showed that both pharmaceutically targeting tau phosphorylation and genetic manipulation of tau could prevent cognitive dysfunction after experimental TBI [17]. We and others have also demonstrated that astrocytic upregulation of IGF-1 after brain injury can improve neurological outcomes in animal models by decreasing phosphor-tau levels [13]. The relationship between mitophagy and tau in TBI sessions remains elucidated. This is consistent with a previous study showing that both impaired mitophagy and p-tau form a vicious circle to aggravate neurodegeneration by affecting each other [38]. In this study, we found that KA-treated neurons had increased mitophagy levels and decreased mitochondrial membrane potential (JC-10 staining) and autophagosomes (p62 expression), while cocultured astrocytes reversed these effects in an IGF-1 pathway-dependent manner. These findings are consistent with previous studies [39, 40]. Importantly, IGF-1 affects both pathways and improves neurological outcome after brain injury. We previously showed that astrocytic IGF-1 could improve the motor and cognitive dysfunction assessed by Y-Maze in KA injured rodents [12]. In this study, we applied the Morris water maze to assess the cognition in TBI mice and found that astrocytic IGF-1 could reduce the cognitive impairment as well and the path tracing demonstrated the learning procedure in these rodents (Supp Figure 13). In addition, in our proteomics study, we also found that TBI reduced the expression of ATIF1 (a mitochondrial ATPase inhibitor) and the ATPase delta subunit, while astrocytic IGF-1 reversed these effects (unpublished data). ATPase inhibitor, mitochondrial (ATIF1) is reported to be a risk factor in Alzheimer's disease (AD) patients with decreased ATIF1 expression in a single-cell seq study [41]. This might explain the mechanism by which mitophagy is impaired after TBI. In our in vitro study, we also found that the facilitation of mitophagy induced by IGF-1 is partly abolished by an IGF-1R antagonist, PI-3K inhibitor, and p38 blocker. This indicates that the impaired mitophagy is due to the dysregulated IGF-1-PI3K-NF-*κ*B pathway. We found that TBI can activate the NF-KB pathway and that astrocytic IGF-1 deactivates NF-*κ*B by KEGG pathway analysis (Supp figure 3). mTOR inhibitors have also been found to increase mitophagy in TBI by activating the IGF-1-PI3K signaling pathway. Meanwhile, after a prediction from the Autophagy Regulatory Network, we found that there might be a relationship between Nfkb1 and Dcp2 (Supp figure 8). This transcriptional regulation of protein-DNA association was also predicted from the JASPAR database (Supp figure 7), and the correlational study between DCP2 and NFKB1 from GEPIA Tool showing the expression in the hippocampus showed that they have a positive correlation with an *R* value at 0.69 (http://gepia.cancer-pku.cn/detail.php) further confirmed this (Supp figure 8). In our findings, Dcp2 increased in excitotoxic neurons, and cocultured astrocytes reversed this (Figure 3). This effect was partly abolished by the IGF-1R antagonist, PI3K inhibitor, and p38 inhibitor. We further performed a chip-qPCR and luciferase assay report to demonstrate the transcriptional regulation effect between NFKB1 and DCP2 (Supp figure 8). Therefore, it is confirmed that the NF-KB pathway might regulate the transcription of Dcp2, and the role of this regulation in the TBI session needs to be validated in future studies.

### 4.4. IGF-1 and IGF-1R Orchestrate Mitophagy through the m7G Pathway as a Novel Regulator of miRNA

A series of mechanisms are involved in cell proliferation, and one of the most critical pathways is the PI3k-AKT signal [42]. Notably, METTL1, one of the major m7G decapping enzymes, can be phosphorylated by AKT directly, and its activity is inhibited aftermost [43]. Considering our findings, it can be predicted that the hypofunction of AKT signal in TBI could increase the levels of m7G-containing miRNAs, like the let-7 family. Particularly, this family suppresses the proliferation of tumors, like breast and lung cancer, by regulating the expression of critical oncogenes including RAS, MYC, and HMGA2 [44]. The control of let-7 family members by the m7G pathway may represent a common mechanism to modulate their expression and activity. Beyond cancer, let-7 is also implicated in neurodegenerative diseases, such as Alzheimer's disease, in which it is significantly upregulated [45]. In addition, reduced let-7 has been found to promote tissue repair by reprogramming cellular metabolism [46, 47]. Therefore, direct targeting of IGF-1/IGF-1R could represent a valid and unexplored therapeutic strategy in these pathological contexts. This report identifies IGF-1 through the m7G pathway as a novel regulator of miRNA function and further affects IGF-1R to orchestrate mitophagy after brain insults. Considering the interest in miRNA and lncRNA targets and tools in therapeutic intervention [48], our findings could be exploited in many noncoding RNA-related disease settings to open up new therapeutic avenues (Supp Figure 12).

Mir-let-7 is found to increase Toll-like receptor 7 and causes neurodegeneration. Specifically, Lehmann et al. applied let-7b into the CSF of WT mice by intrathecal injection resulted in neurodegeneration [49]. Accordingly, let-7 is increased in CSF of patients with AD, which is consistent with our finding and demonstrates a neurotoxic effect [50]. In addition, in a recent study regarding the plasma exosome, Nie et al. found that plasma exo-miR-let-7e is decreased in AD patients [51]. When we applied miR-let-7e antagonist in TBI mice, this antagonist could prevent neurodegeneration in TBI, as reflected by HE staining, ultrastructure of hippocampal tissue by an electron microscopy, and Nissl's staining. And this neuroprotective effect of let-7e inhibitor is partly blocked by AG1024 (IGF-1R inhibitor) and further rescued by astrocytic IGF-1. This indicates that IGF-1 and IGF-1R orchestrate mitophagy and neuroprotection following TBI via miR-let-7e, and exosomal let-7e might be a biomarker to assess the brain severity and recovery progress. IGF-1 has multiple sources, including neurons, astrocytes, and microglia. It was previously found that microglial activation could influence the growth factor expression, and neuroinflammation was able to cause neurodegeneration by suppressing the production of microglia-derived IGF-1 [52]. A recent study also shows that microglia retain the ability to express IGF-1 in the aged mice [53]. However, a recent study demonstrated that microglia is a dominant target of IGF-1 in the stroke mice. The subcutaneous injection of IGF-1 is able to increase the expression of microglia in the brain [54]. Therefore, the exact relationship of IGF-1 among the neurons, astrocytes, and microglia needs to be explored in future study with 3D culture and organoids, which is more relevant to the real situation.

An earlier version of it has been presented as “preprint” in “Research Square” according to the following link: https://www.researchsquare.com/article/rs-41602/v15 [55].

## 5. Conclusions

In our study, we found that astrocytic IGF-1 regulates miR-let-7e through DCP2 and further affects IGF-1R to orchestrate mitophagy in TBI via exosomal let-7e. Astrocytic IGF-1/IGF-1R might affect the maturation of noncoding RNAs to facilitate mitophagy and improve neurological outcomes after brain insults. We also demonstrate a direct evidence that IGF-1 could facilitate the mitophagy via adjusting exosomal secretion and its contents. This suggests that IGF-1-engineered exosome might be a novel therapeutic method for TBI sessions.

## Figures and Tables

**Table 1:** Patient characteristics by IGF-I group.

Characteristic	IGF-1 above median group	IGF-1 below median group	*p* value
No.	132	97	na
Age at onset (years)	41.3 ± 14.5	39.6 ± 12.1	0.3487
Male, no.	92	61	0.3208
Moderate-severe TBI	14	30	0.0002
Time since TBI (hours)	5.3 ± 2.2	5.5 ± 3.1	0.5685
Absolute IGF-1 (nmol/l)	175.18 ± 71.86	131.22 ± 57.15	<0.0001
Any pituitary deficiency, no.	11	19	0.0168
Any structural brain abnormality, no.	66	76	<0.0001
No. of contusions	42	68	<0.0001
Diffuse axonal injury, no.	5	8	0.1618
GCS	12.2 ± 2.1	9.3 ± 3.9	<0.0001
GOS	4.7 ± 1.7	3.2 ± 2.2	<0.0001

## Data Availability

The dataset supporting the conclusions of this article are available from the corresponding author.

## Supplementary Materials

Supplementary Materials Supp Figure 1: single cell RNA sequencing data in TBI. (A) Cell cluster by UMAP in TBI. (B, C) Marker gene CST7 and IGF-1 expression in different clusters. Supp Figure 2: cell-cell interaction based on IGF-1 in TBI sc-RNA-seq. (A–D) CellChat analysis shows that several clusters have an association based on IGF-1-IGF1R. (E–H) Monocle analysis demonstrates the IGF-1 in pseudotime results. (I) Violin map shows the expression of Igf1, Igf2, and Igf1r in different clusters which is consistent with (D) showing clusters 13 and 15 as the main senders and clusters 4, 9, 10, 19, and 20 as the main receivers. Supp Figure 3: KEGG clustering for proteins after TBI and astrocytic IGF-1 treatment. TBI increases p50 (NF-KB) in the brains while astrocytic IGF-1 decreases this (red indicates increase, and cyan indicates decrease). Supp Figure 4: KA induces neuronal loss in vitro. KA-treated neurons show decreased MAP-2 and beta-tubulin expression, while cocultured astrocytes could increase their expression. ^∗^*p* < 0.05, compared to other groups. Supp Figure 5: Dcp2 knockdown in neurons shows altered miRNAs and mRNAs. Mir-let-7e listed at the top downregulated miRNA in Dcp2 knockdown neurons. Left, ratio of Log2 KO to WT, and right is the heat map. Supp Figure 6: astrocytic IGF-1 reduces phosphorylated tau expression (Ser356 fluorescence) in hippocampus (DG) and ipsilateral cortex (Cx) and prevents neuronal death (NeuN) accordingly. TBI reduces the mean density of NeuN staining compared to the sham group, while astrocytic IGF-1 increases the neuronal mean density. The mean fluorescence density of Ser356 also increases in TBI mice, while astrocytic IGF-1 decreases it. ^∗^*p* < 0.05, compared to other groups. *n* = 3 in each group. Supp Figure 7: a prediction from Autophagy Regulatory Network shows a potential relationship between Nfkb1 and Dcp2. Supp Figure 8: Nfkb1 (p50) can activate Dcp2 transcription to regulate miRNAs and downstream pathways. (A) Schematic diagram showing Nfkb1-binding motif (from JASPAR database) and three potential Dcp2 elements (E1, E2, and E3) in the Dcp2 promoter region. TSS is the transcriptional start site of Dcp2. (B) Dcp2 mRNA expression with overexpressed Nfkb1 overexpression in U251 cell line and primary cultured neurons (PCN). (C) Dcp2 mRNA expression when treated with 10 *μ*mol Nfkb1 inhibitor (MG132). (D) Dcp2 mRNA expression when treated with Nfkb1 shRNA. (E) Luciferase activity between Dcp2 promoter and Nfkb1 plasmids (0, 200, and 400 ng). (F) Dcp2 promoter activity when cells treated with 10 *μ*mol Nfkb1 inhibitor. (G) Luciferase activity of Dcp2 promoter when treated with Nfkb1 shRNA. (H) Three predicted binding sites between Nfkb1 and Dcp2 promoter were deleted and named E1-Del, E2-Del, and E3-Del. (H, I) Luciferase activity between three Dcp2 promoter deletion mutants and overexpressed Nfkb1 in U251 cells and primary cultured neurons. (J) ChIP qPCR shows that Nfkb1 binds to the E1 element of Dcp2 promoter. IgG is negative control. The data are presented as the mean ± sem (*n* = 3); ^∗^*p* < 0.05. Supp Figure 9: intensity/size graph for experiment. The figure shows the intensity and size of the exosomes isolated from each group. Q1: exosomes from the primary neuronal culture medium. Q2: exosomes from the coculture neurons with astrocyte medium. Q3: exosomes from the KA-treated primary neuronal culture medium. Q4: exosomes from the KA-treated coculture neurons with astrocyte medium. Supp Figure 10: FTLA concentration/size graph for experiment. The figure shows the intensity and size of the exosomes isolated from each group. Q1: exosomes from the primary neuronal culture medium. Q2: exosomes from the coculture neurons with astrocyte medium. Q3: exosomes from the KA-treated primary neuronal culture medium. Q4: exosomes from the KA-treated coculture neurons with astrocyte medium. Supp Figure 11: representative EM images for exosomes from each group. The mean diameter of exosomes in this experiment is from 50 to 200 nm. The figure shows the intensity and size of the exosomes isolated from each group. Q1: exosomes from the primary neuronal culture medium. Q2: exosomes from the coculture neurons with astrocytes medium. Q3: exosomes from the KA-treated primary neuronal culture medium. Q4: exosomes from the KA-treated coculture neurons with astrocyte medium. Supp Figure 12: astrocytic IGF-1 and IGF-1R orchestrate the mitophagy in TBI. Astrocytic IGF-1 decreases the expression of let-7e and causes disturbances of miRNAs after TBI. Astrocytic IGF-1 and IGF-1R facilitate the mitophagy in TBI through NFKB1-Dcp2-let-7e. Supp Figure 13: representative path tracing in MWM tests for four groups of mice. Supp Figure 14: raw WB blots for Figures 2 and 5. Supplementary Table 1: qPCR primers in this study.
