# Supplementary material for: Astrocytic IGF-1 and IGF-1R Orchestrate Mitophagy in Traumatic Brain Injury via Exosomal miR-let-7e
Source: Oxid Med Cell Longev. 2022 Aug 24;2022:3504279. doi: 10.1155/2022/3504279 (PMC9433209; doi:10.1155/2022/3504279)
Supplement: Supplementary Materials — Supp Figure 1: single cell RNA sequencing data in TBI. (A) Cell cluster by UMAP in TBI. (B, C) Marker gene CST7 and IGF-1 expression in different clusters. Supp Figure 2: cell-cell interaction based on IGF-1 in TBI sc-RNA-seq. (A–D) CellChat analysis shows that several clusters have an association based on IGF-1-IGF1R. (E–H) Monocle analysis demonstrates the IGF-1 in pseudotime results. (I) Violin map shows the expression of Igf1, Igf2, and Igf1r in different clusters which is consistent with (D) showing clusters 13 and 15 as the main senders and clusters 4, 9, 10, 19, and 20 as the main receivers. Supp Figure 3: KEGG clustering for proteins after TBI and astrocytic IGF-1 treatment. TBI increases p50 (NF-KB) in the brains while astrocytic IGF-1 decreases this (red indicates increase, and cyan indicates decrease). Supp Figure 4: KA induces neuronal loss in vitro. KA-treated neurons show decreased MAP-2 and beta-tubulin expression, while cocultured astrocytes could increase their expression. ∗p < 0.05, compared to other groups. Supp Figure 5: Dcp2 knockdown in neurons shows altered miRNAs and mRNAs. Mir-let-7e listed at the top downregulated miRNA in Dcp2 knockdown neurons. Left, ratio of Log2 KO to WT, and right is the heat map. Supp Figure 6: astrocytic IGF-1 reduces phosphorylated tau expression (Ser356 fluorescence) in hippocampus (DG) and ipsilateral cortex (Cx) and prevents neuronal death (NeuN) accordingly. TBI reduces the mean density of NeuN staining compared to the sham group, while astrocytic IGF-1 increases the neuronal mean density. The mean fluorescence density of Ser356 also increases in TBI mice, while astrocytic IGF-1 decreases it. ∗p < 0.05, compared to other groups. n = 3 in each group. Supp Figure 7: a prediction from Autophagy Regulatory Network shows a potential relationship between Nfkb1 and Dcp2. Supp Figure 8: Nfkb1 (p50) can activate Dcp2 transcription to regulate miRNAs and downstream pathways. (A) Schematic diagram showing Nfkb1-binding mot [file 3504279.f1.pdf]

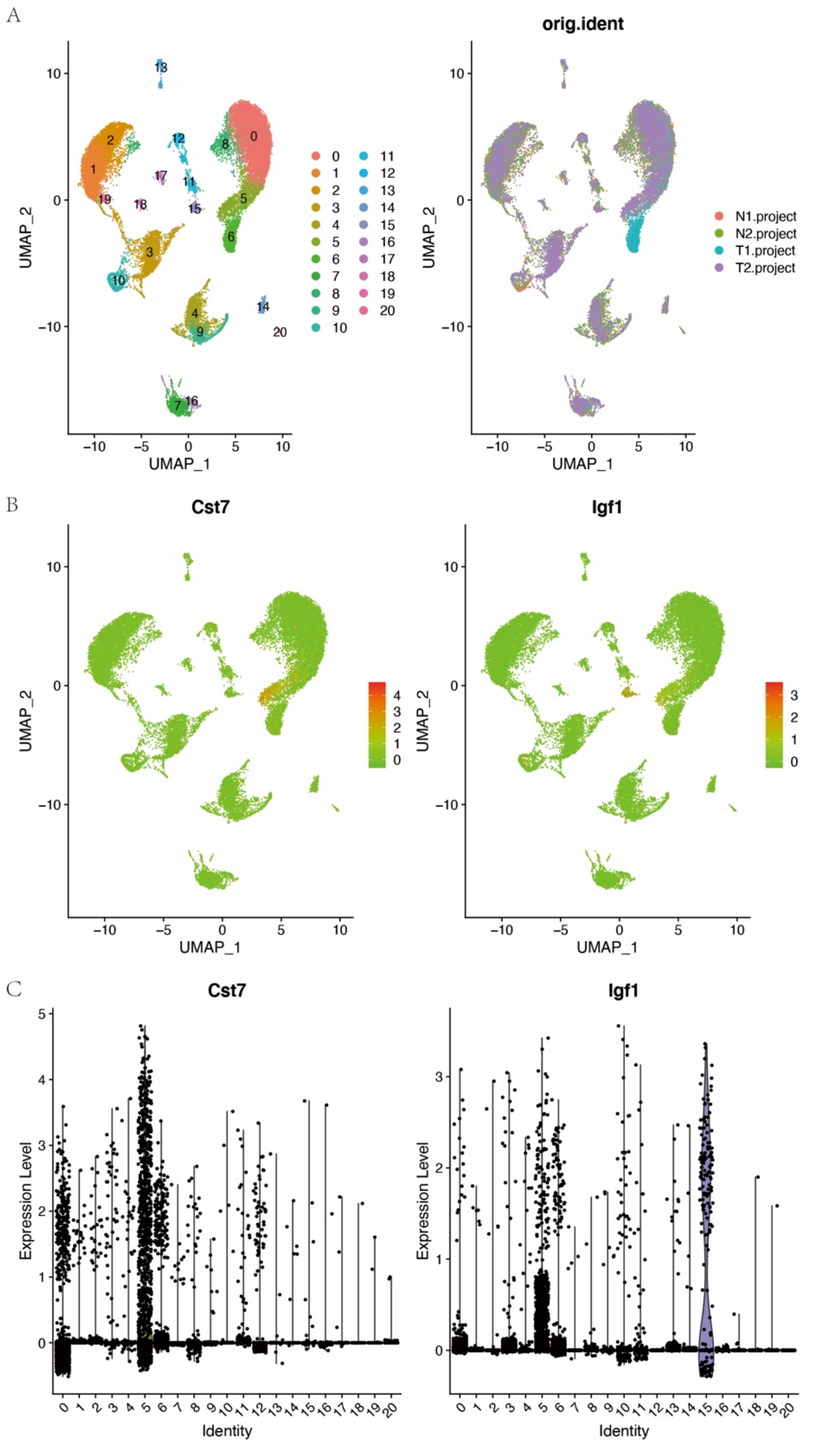

Supp Fig 1. Single cell RNA sequencing data in TBI. A. Cell cluster by UMAP in TBI. B&C. Marker gene CST7 and IGF-1 expression in different clusters.

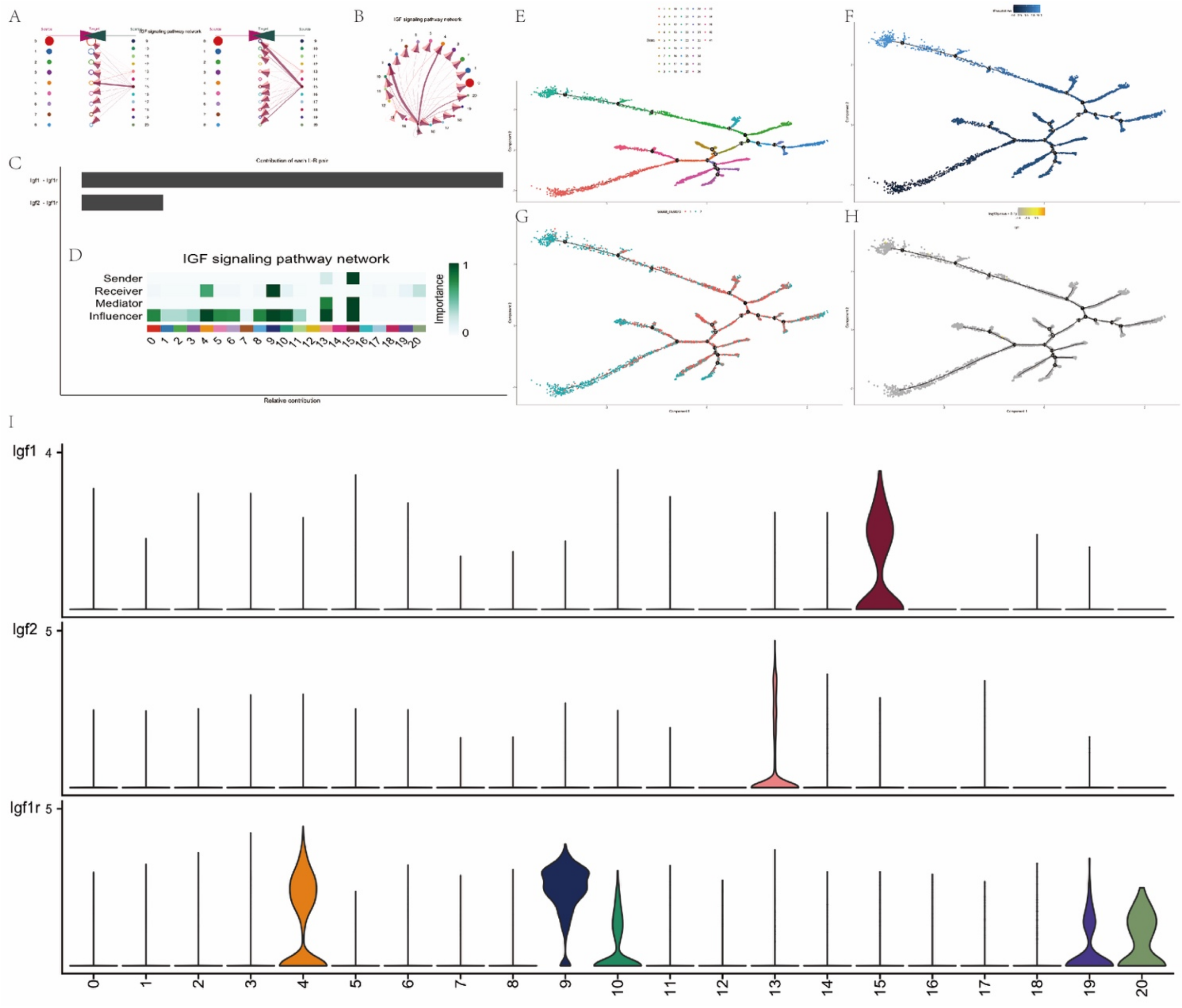

Supp Fig 2. Cell-cell interaction based on IGF-1 in TBI sc-RNA seq. A-D. CellChat analysis shows several clusters have an association based on IGF-1-IGF1R.E-H. Monocle analysis demonstrates the IGF-1 in pseudotime results. I. Violin map shows the expression of Igf1, Igf2 and Igf1r in different clusters which is consistent with Figure D showing cluster 13 and 15 as the main senders and cluster 4,9,10,19 and 20 as the main receivers.

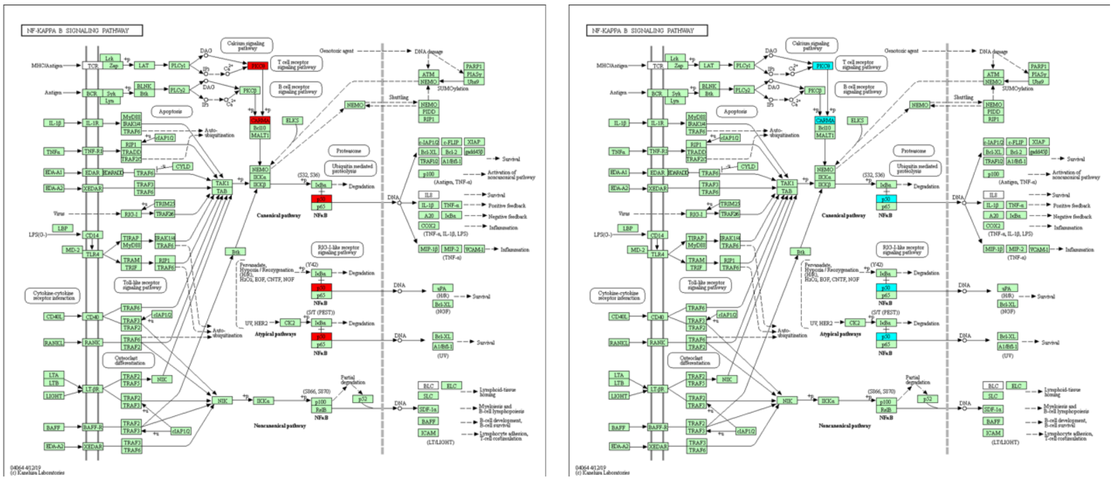

Supp Fig 3. KEGG clustering for proteins after TBI and astrocytic IGF-1 treatment. TBI increases p50 (NF-KB) in brains while astrocytic IGF-1 decreases this (red indicates increase and cyan indicates decrease).

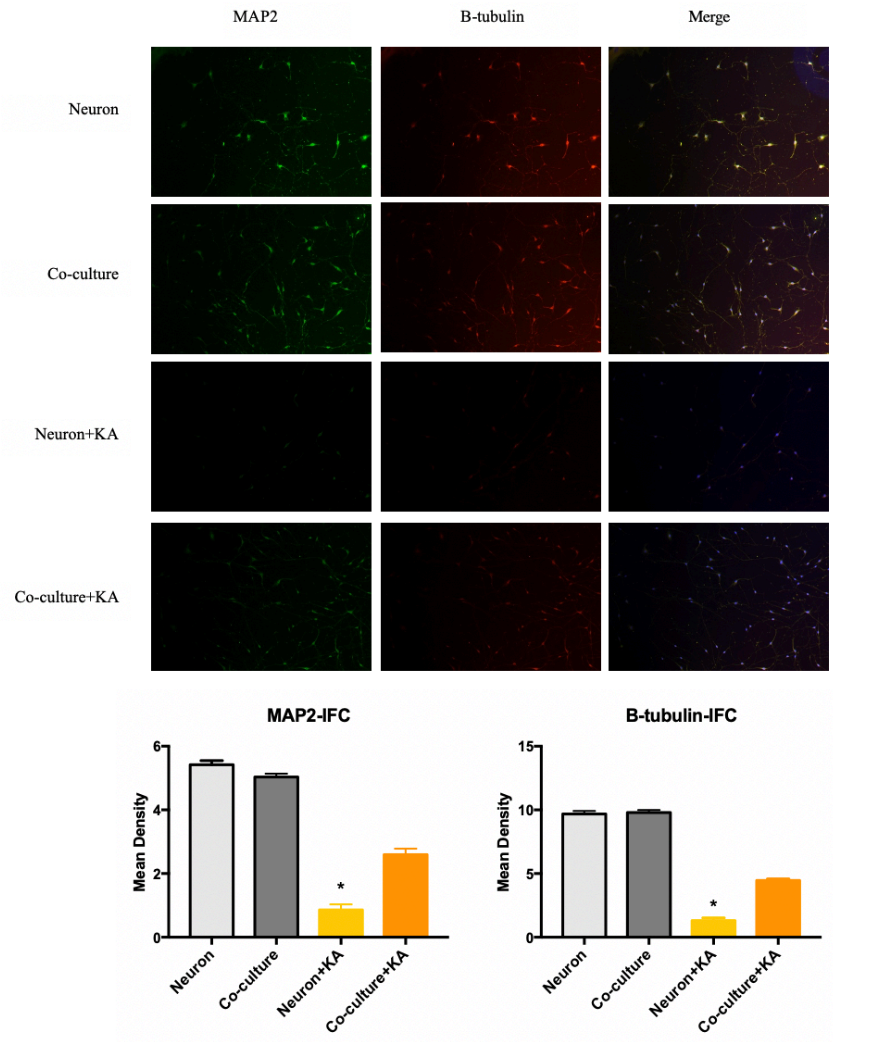

Supp Fig 4. KA induces neuronal loss in-vitro. KA treated neurons show decreased MAP-2 and beta-tubulin expression; while cocultured astrocytes could increase their expression. * p<0.05, compared to other groups.

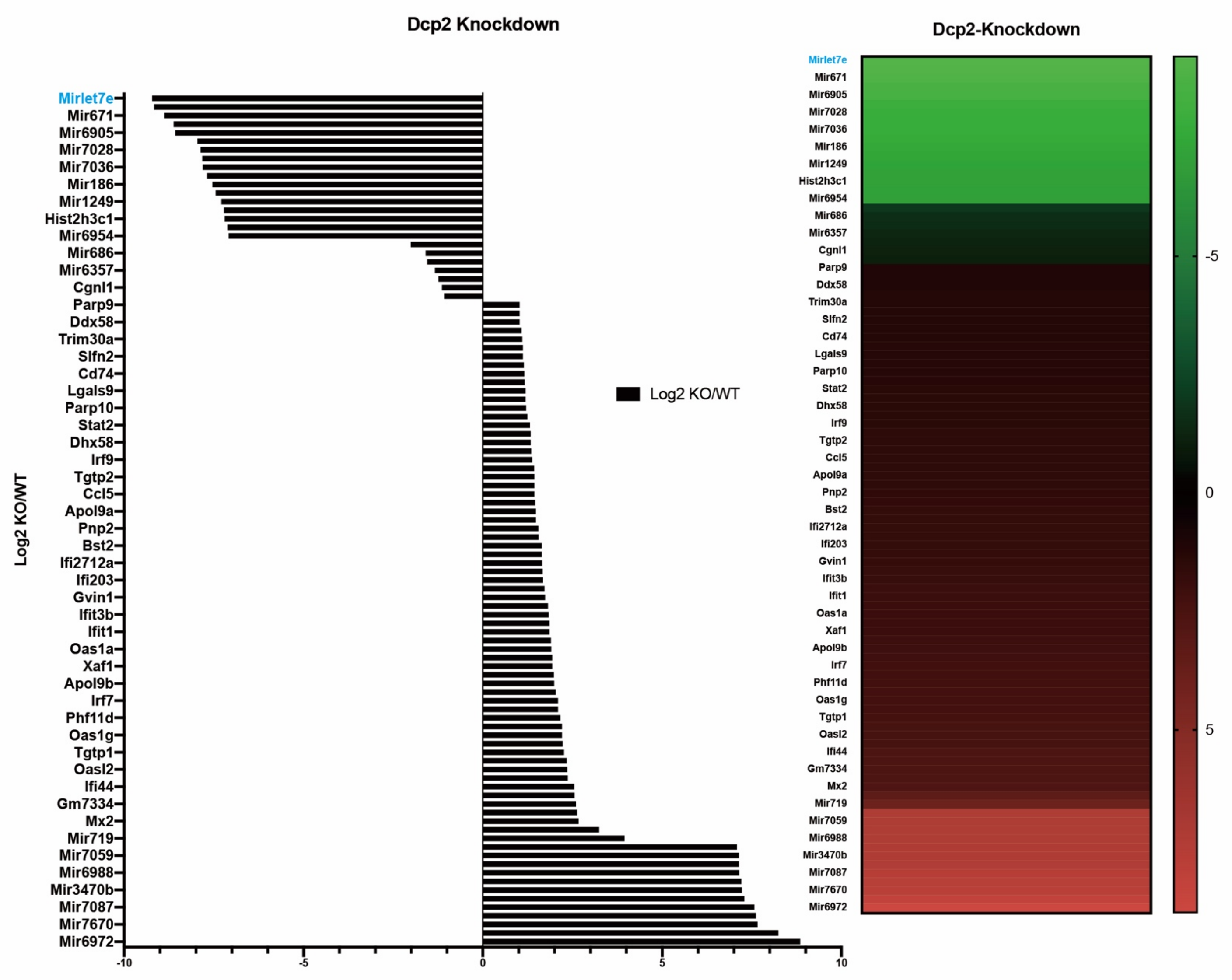

Supp Fig 5. Dcp2 knockdown in neurons shows altered miRNAs and mRNAs. Mir-let-7e listed at the top down-regulated miRNA in Dcp2 knockdown neurons. Left, ratio of Log2 KO to WT, and right is the heatmap.

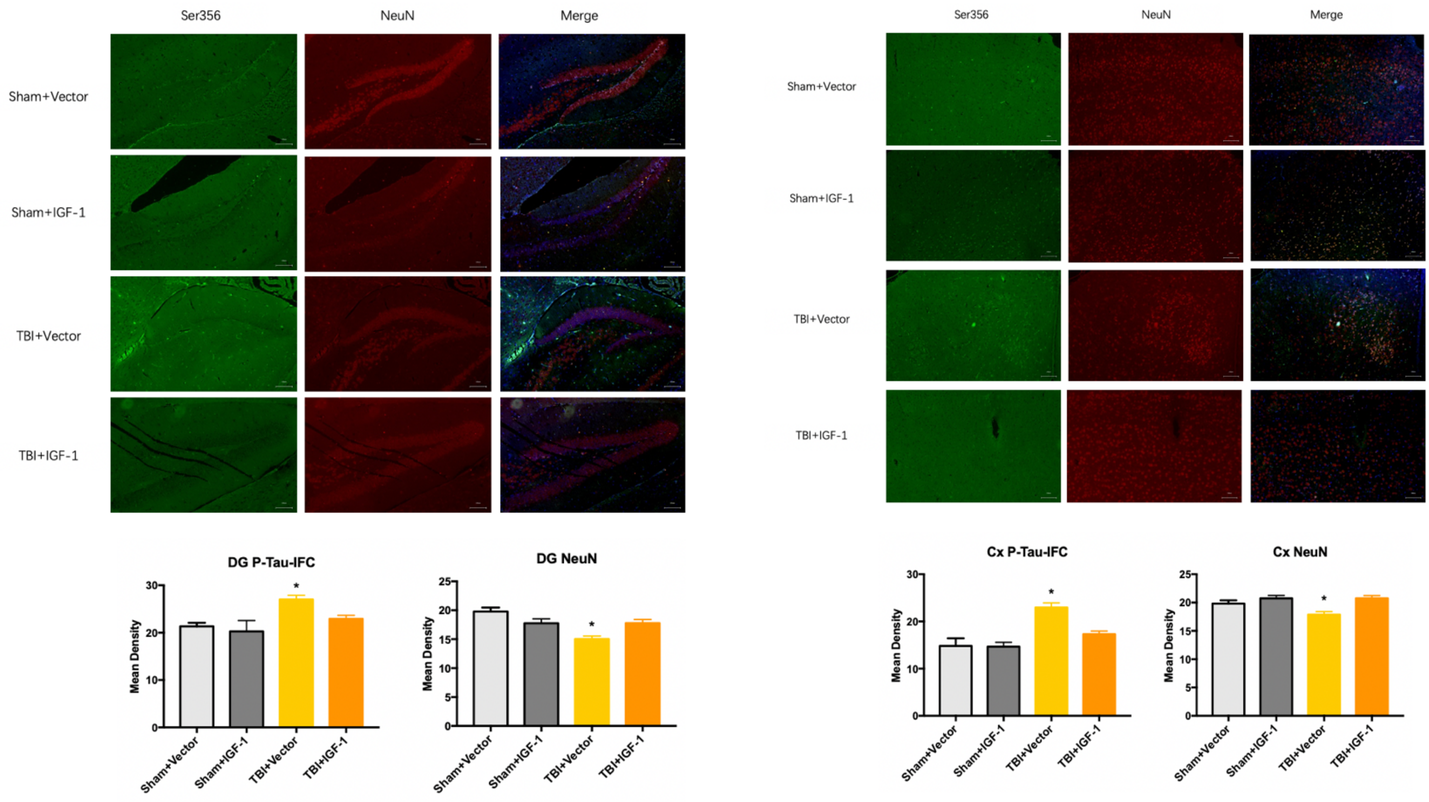

Supp Fig 6. Astrocytic IGF-1 reduces phosphorylated tau expression (Ser356 fluorescence) in hippocampus (DG) and ipsilateral cortex (Cx) and prevents neuronal death (NeuN) accordingly. TBI reduces the mean density of NeuN staining compared to sham group, while astrocytic IGF-1 increases the neuronal mean density. The mean fluorescence density of Ser356 also increases in TBI mice, while astrocytic IGF-1 decreases it. $*p < 0.05$, compared to other groups. n = 3 in each group.

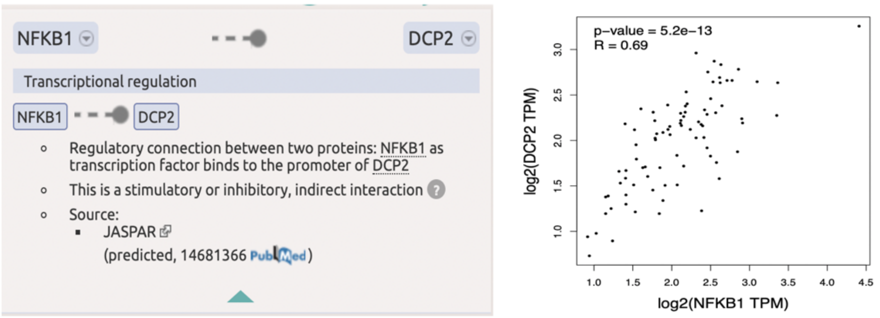

Supp Fig 7. A prediction from Autophagy Regulatory Network shows a potential relationship between Nfkb1 and Dcp2.

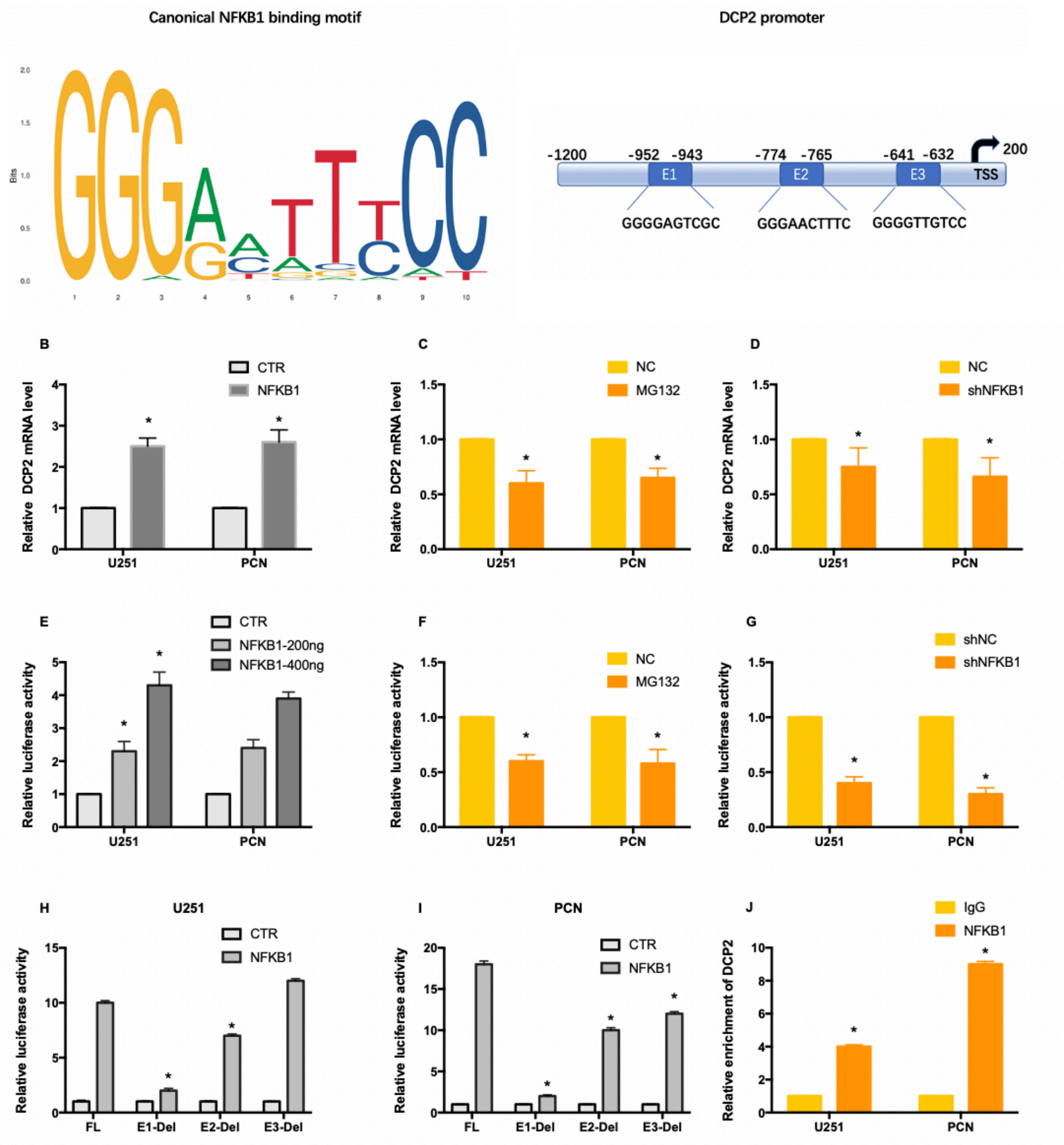

Supp Fig 8. Nfkb1 (p50) can activate Dcp2 transcription to regulate miRNAs and downstream pathways. A. Schematic diagram showing Nfkb1-binding motif (from JASPAR Database) and three potential Dcp2 elements (E1, E2, and E3) in the Dcp2 promoter region. TSS is the transcriptional start site of Dcp2. B. Dcp2 mRNA expression with overexpressed Nfkb1 overexpression in U251 cell line and primary cultured neurons (PCN). C. Dcp2 mRNA expression when treated with 10 µmol Nfkb1 inhibitor (MG132). D. Dcp2 mRNA expression when treated with Nfkb1 shRNA. E. Luciferase activity between Dcp2 promoter and Nfkb1 plasmids (0, 200, and 400 ng). F. Dcp2 promoter activity when cells treated with 10 µmol Nfkb1 inhibitor. G. Luciferase activity of Dcp2 promoter when treated with Nfkb1 shRNA. H Three predicted binding sites between Nfkb1 and Dcp2 promoter was deleted and named E1-Del, E2-Del, and E3-Del. H & I. Luciferase activity between three Dcp2 promoter deletion mutants and overexpressed Nfkb1 in U251 cells and primary cultured neurons. J. ChIP qPCR shows that Nfkb1 binds to the E1 element of Dcp2 promoter. IgG is negative control. The data are presented as the mean ± sem. (n

= 3); *P < 0.05.

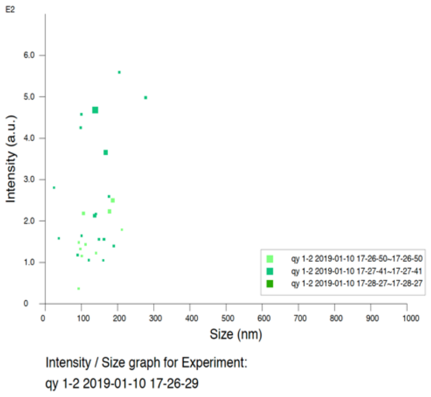

Q1

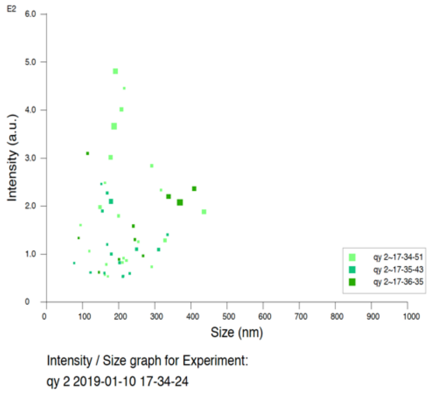

Q2

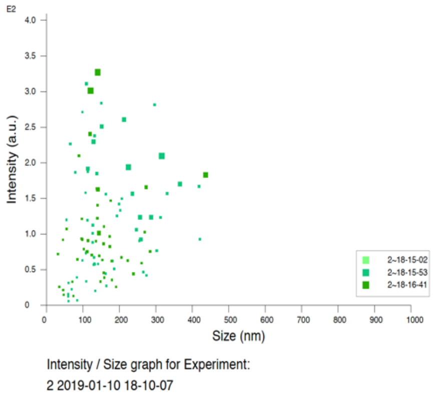

Q3

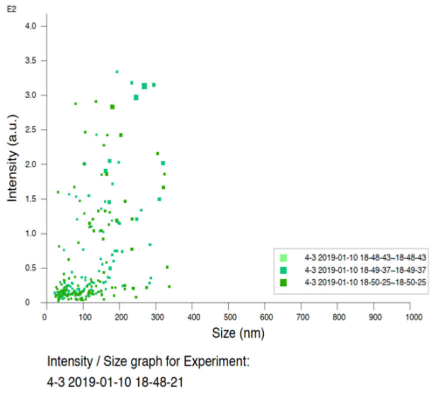

Q4

Supp Fig 9. Intensity / Size graph for Experiment. The figure shows the intensity and size of the exosomes isolated from each group. Q1: Exosomes from the primary neuronal culture medium. Q2: Exosomes from the coculture neurons with astrocytes medium. Q3: Exosomes from the KA treated primary neuronal culture medium. Q4: Exosomes from the KA treated coculture neurons with astrocytes medium.

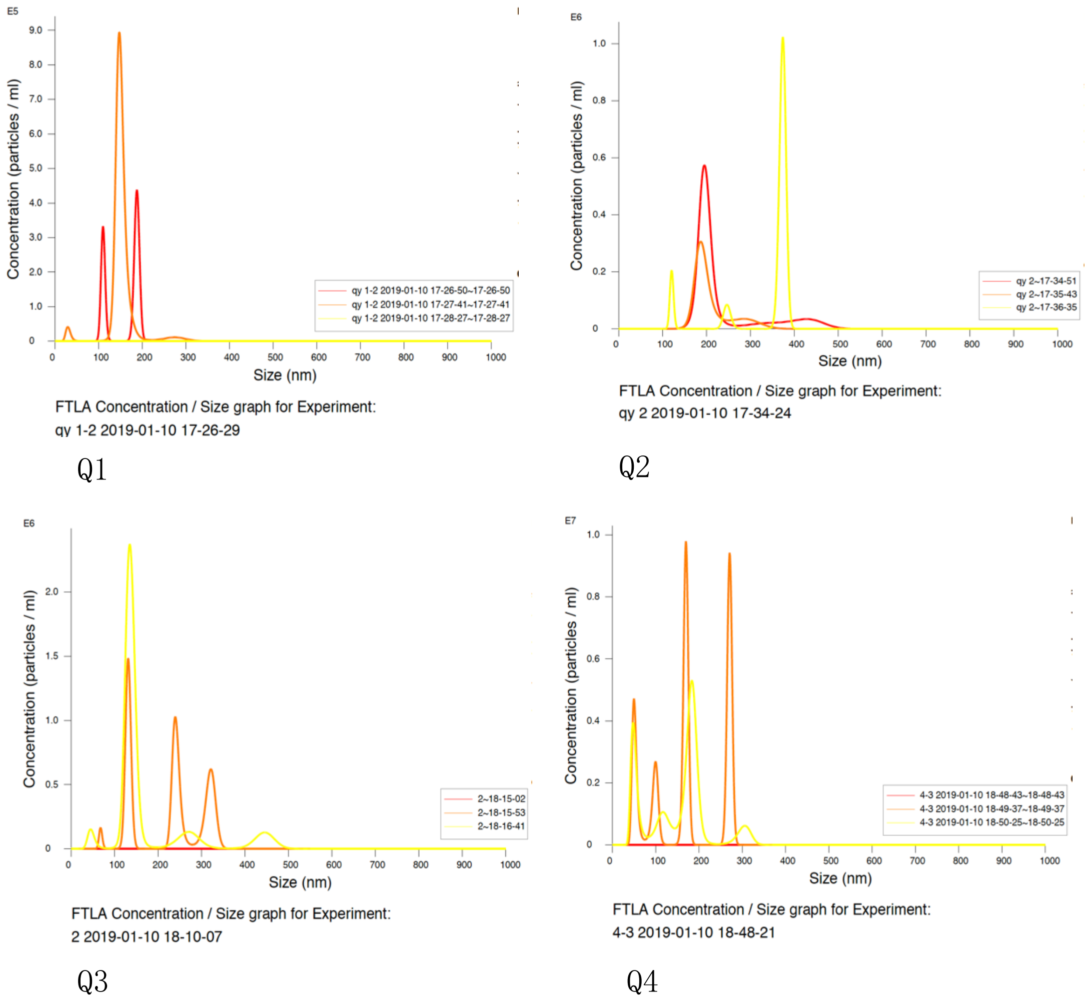

Supp Fig 10. FTLA concentration/ Size graph for Experiment. The figure shows the intensity and size of the exosomes isolated from each group. Q1: Exosomes from the primary neuronal culture medium. Q2: Exosomes from the coculture neurons with astrocytes medium. Q3: Exosomes from the KA treated primary neuronal culture medium. Q4: Exosomes from the KA treated coculture neurons with astrocytes medium.

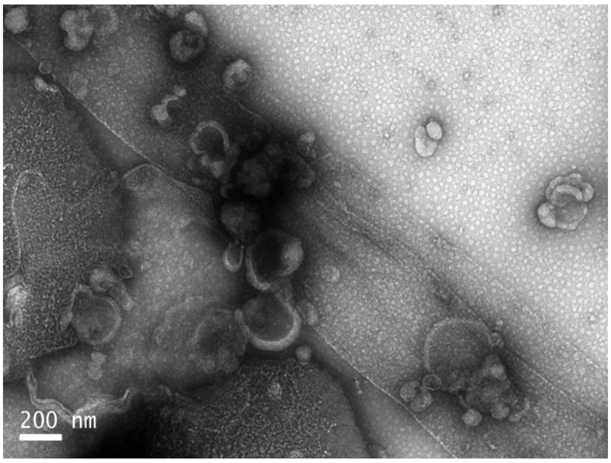

Q1

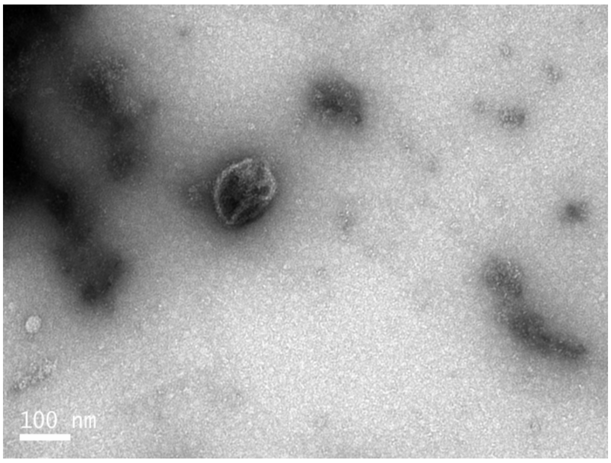

Q2

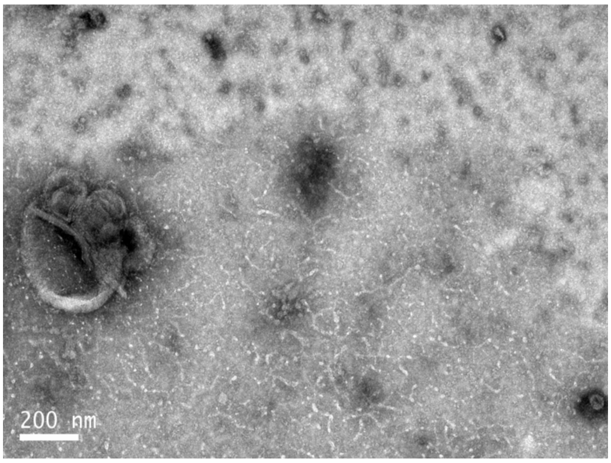

Q3

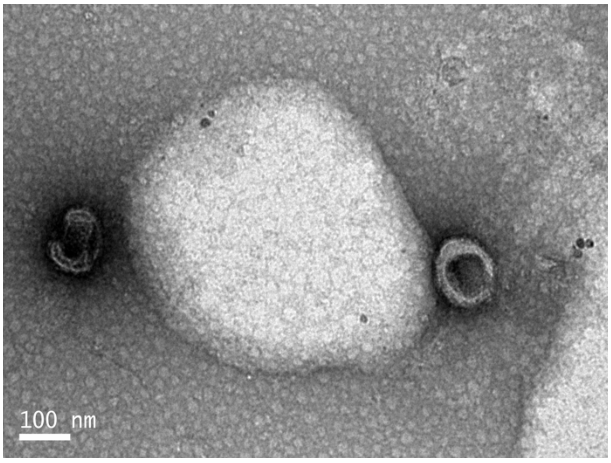

Q4

Supp Fig 11. Representative EM images for exosomes from each group. The mean diameter of exosomes in this experiment is from 50-200nm. The figure shows the intensity and size of the exosomes isolated from each group. Q1: Exosomes from the primary neuronal culture medium. Q2: Exosomes from the coculture neurons with astrocytes medium. Q3: Exosomes from the KA treated primary neuronal culture medium. Q4: Exosomes from the KA treated coculture neurons with astrocytes medium.

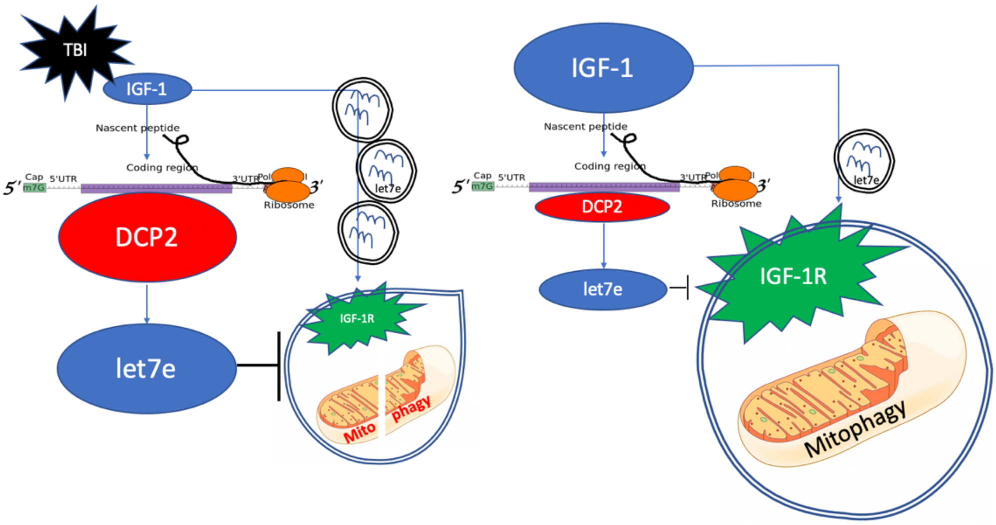

Supp Fig 12. Astrocytic IGF-1 and IGF-1R orchestrate the mitophagy in TBI. Astrocytic IGF-1 decreases the expression of let 7e and cause disturbances of miRNAs after TBI. Astrocytic IGF-1 and IGF-1R facilitate the mitophagy in TBI through NFKB1-Dcp2-let 7e.

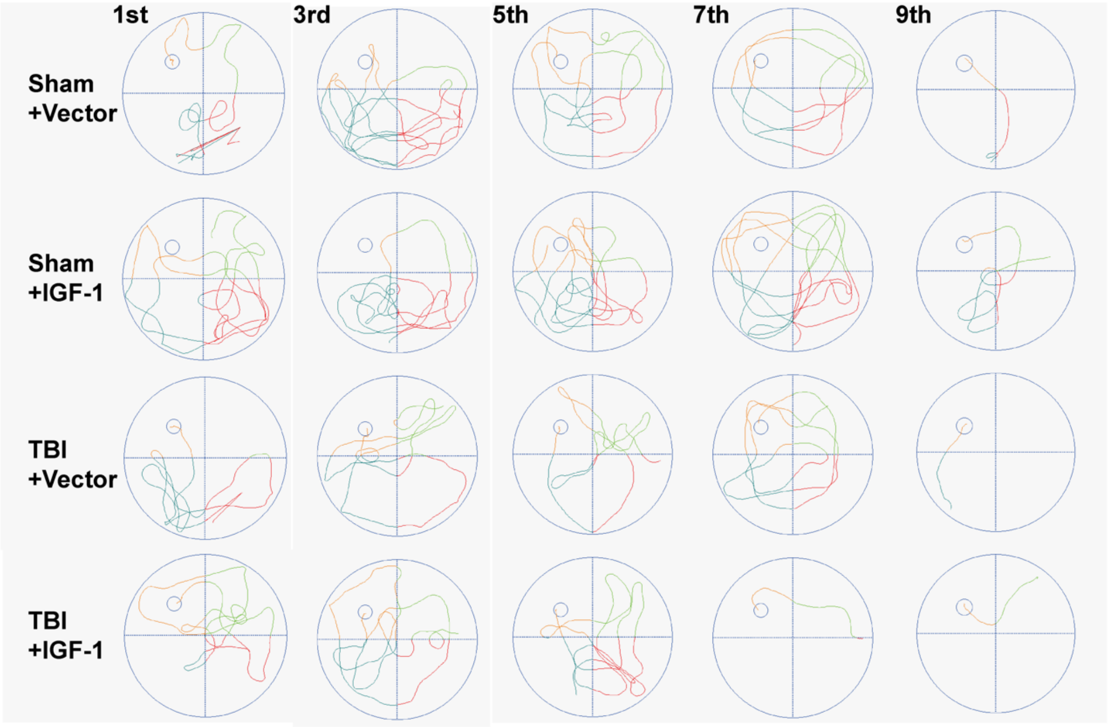

Supp Fig 13. Representative path tracing in MWM tests. 1st, 3rd, 5th, 7th, 9th was the day of the MWM test.

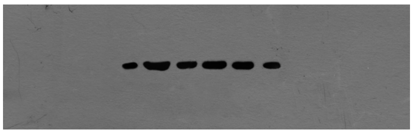

LC3-Atg8

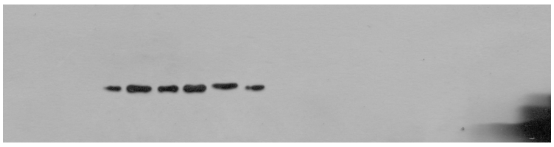

Atg5

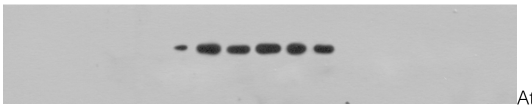

Atg14

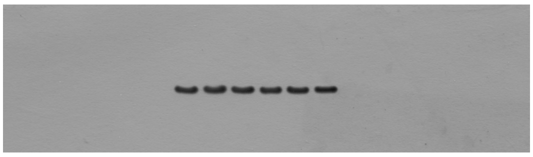

β-actin

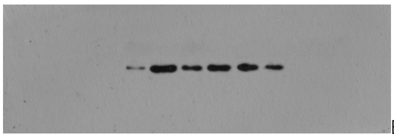

Beclin-1

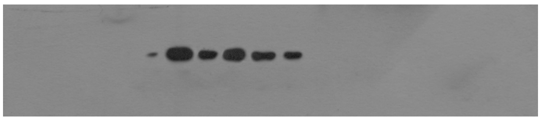

Lamps

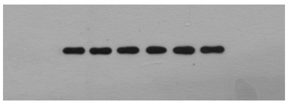

β-actin2

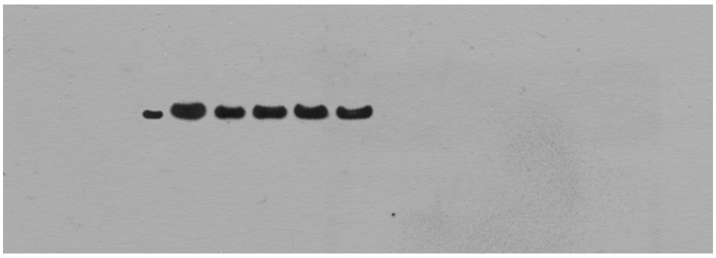

DCP2

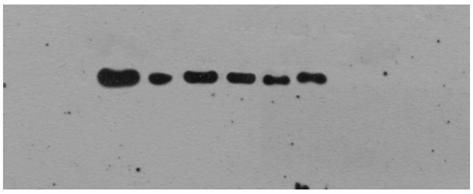

IGF-1R

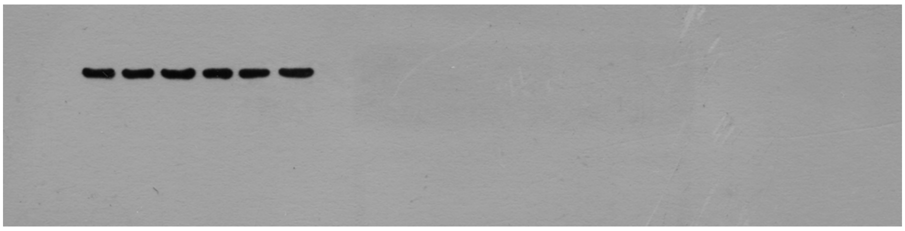

β-actin

Supp Fig 14. Raw WB blots for Figure 5 and Figure 3.

Supp Table 1.

qPCR Taqman Primers		Sequence
18sRNA	FW	TGTGCCGCTAGAGGTGAAATT
18sRNA	Rev	TGGCAAATGCTTTCGCTTT
U6	FW	CTCGCTTCGGCAGCACA
U6	Rev	AACGCTTCACGAATTTGCGT
Let-7e	FW	GGGCTGAGGTAGGAGGTTGT
Let-7e	Rev	GGGAAAGCTAGGAGGCCGTA
Dcp2	FW	AGACAATGCGATCCGAGTGTG

Dcp2	Rev	CGTAAGTCGGGACTCCCATTT
Nfkb1	FW	GGCAGCACTACTTCTTGACC
Nfkb1	Rev	CAGCAAACATGGCAGGCTAT

qPCR Taqman Advanced miRNA Assay Ids (Thermo Fisher Cat# A25576)

hsa-let-7e-5p 478579_mir

CHIP-PCR Primers

DCP Promoter E1	GGGGAGTCGC
DCP Promoter E2	GGGAACTTTC
DCP Promoter E3	GGGGTTGTCC
